# Seagrasses are most vulnerable to marine heatwaves in tropical zones: local‐scale and broad climatic zone variation in thermal tolerances

**DOI:** 10.1111/nph.70742

**Published:** 2025-12-02

**Authors:** Nicole Said, Chanelle Webster, Simone Strydom, Natasha Dunham, Matthew P. Adams, Kathryn McMahon

**Affiliations:** ^1^ Centre for Marine Ecosystems Research, School of Science Edith Cowan University Joondalup WA 6027 Australia; ^2^ Marine Science Program, Biodiversity and Conservation Science, Department of Biodiversity Conservation and Attractions Kensington WA 6151 Australia; ^3^ School of Mathematical Sciences and Centre for Data Science Queensland University of Technology Brisbane Qld 4000 Australia; ^4^ Securing Antarctica's Environmental Future Queensland University of Technology Brisbane Qld 4000 Australia; ^5^ Centre for People, Place and Planet Edith Cowan University Joondalup WA 6027 Australia

**Keywords:** climate change, foundation species, ocean warming, photo‐physiology, physiology, plants, resilience, seagrass

## Abstract

- Under a changing climate, it is imperative that we understand how species may respond to temperature impacts, which can differ among populations of the same species due to local drivers. Thermal tolerance data, which can be used to assess an organism's upper thermal limits, is valuable to identify species and/or populations' susceptibility to thermal stress.
- This study assessed the variation in thermal tolerance of six seagrass species at both broad latitudinal (*c*. 500–1000 km) and local scales (*c*. 25 km). Photosynthesis–temperature curves (15–42°C) were conducted, by measuring oxygen in closed incubation chambers, and thermal optima (*T*_opt_) was extracted. [Correction added on 19 January 2026, after first online publication: the temperature range in the preceding sentence has been corrected.]
- We found that *T*_opt_ varied by almost 10°C among six species, and *T*_opt_ for the same species differed by up to 4°C across both broad and local scales, but with no consistent patterns across latitude. This highlights that thermal performance does not necessarily reflect thermal geography of a seagrass species range, and that other environmental variables may play a role in how species respond to temperature.
- Overall, while some seagrass species may benefit from small increases in temperature, marine heatwaves are likely to have negative implications for five of the six species assessed, with greater impacts occurring in tropical regions.

## Abstract 2

See also the Commentary on this article by Lawrence, **249**: 2603–2605.

## Introduction

Global temperature changes are a primary driver of biodiversity loss, with climate‐related impacts causing unprecedented ecological consequences in marine ecosystems (Halpern *et al*., 2019; Trisos *et al*., 2020). As marine heatwaves (MHWs) become more intense and frequent world‐wide (Oliver *et al*., 2018), ecological ramifications, including mass die‐offs, range shifts, alterations in community structure and reduced performance, are becoming increasingly evident as species encounter temperatures beyond their thermal tolerance – the range of temperatures in which an organism can function (Wiens, 2016; Feeley *et al*., 2020; Strydom *et al*., 2020). Sessile habitat‐forming organisms, such as plants, are particularly vulnerable to these extreme temperature events (Corlett & Westcott, 2013). To date, the majority of plant thermal tolerance studies have focused on terrestrial crop species of economic importance (Geange *et al*., 2021), reflecting significant investment dedicated to enhancing agricultural productivity and demonstrating the critical value of thermal tolerance data (Corlett & Westcott, 2013). Thermal tolerance data can also be used to predict how ecologically important marine plant species respond to climate‐induced increases in temperature and can identify species vulnerability to climate impacts (Bennett *et al*., 2022a,b). This information for a species at the population level can be used to inform resilience‐building opportunities, such as assisted gene flow if population‐specific differences in thermal tolerance exist (Gaitán‐Espitia & Hobday, 2020; Coleman & Bragg, 2021).

As the globe heats up, the resilience and persistence of plants, in part, depends on a species' upper physiological thermal tolerance limits, plasticity (ability to acclimate) and evolutionary adaptations (particular genes under selection that provide an individual with an advantage over generations can become more common; Quigley, 2024; Somero, 2010). It is widely accepted that a species' geographic range is not constrained by temperature alone, but rather, is constrained by a suite of environmental conditions (e.g. dispersal ability and soil properties) in which a species can grow and reproduce; known as a species' realised niche (Bennett *et al*., 2018; Bates & Bertelsmeier, 2021). If a species' physiological thermal tolerance (fundamental thermal niche) is higher than a species' realised thermal niche, species may be afforded short‐term resilience, or may be able to acclimate to climate‐induced temperature impacts ‘to persist in place’ or, even be positively influenced, by exhibiting an increase in productivity (Bennett *et al*., 2018). If a species' physiological thermal tolerance is lower than predicted temperature increases, species may be negatively impacted (e.g. reduced productivity or mortality; Wooliver *et al*., 2022). Experimentally derived thermal tolerance data (usually through a thermal performance curve (TPC)) can be used to assess which species have realised niches that are closer to their upper physiological thermal tolerances and, therefore, may be more vulnerable to climate change (Bennett *et al*., 2018).

Thermal tolerance parameters and measures of biological rate processes (e.g. productivity) can be mathematically extracted from TPCs (Adams *et al*., 2017; Wooliver *et al*., 2022). Common TPCs in plant studies are those which assess the relationship between photosynthesis and temperature (*P*–*T*). *P*–*T* curves typically show a gradual increase in photosynthetic rates with increased temperature to a thermal optimum (*T*_opt_), *T*_opt_ being where net photosynthesis is highest (NP_max_), followed by a steep decline past *T*_opt_ to a species critical thermal maxima (CT_max_; Wooliver *et al*., 2022). The ecological relevance of these temperature tolerance metrics (*T*_opt_ and CT_max_) can vary. For example, CT_max_ typically exceeds temperatures experienced within a species habitat, and most physiological studies indicate that CT_max_ represents an acute lethal limit, with *c*. 50% mortality occurring within minutes to hours of exposure (Verberk *et al*., 2023). Therefore, CT_max_ indicates temperatures at which rapid, widespread mortality is likely to occur and is useful for assessing broad scale thermal tolerance plasticity among species, or populations; however, it does not capture other ecologically relevant impacts, such as declines in productivity or partial mortality (Verberk *et al*., 2023; Dichiera *et al*., 2024). We suggest *T*_opt_ has greater ecological relevance (than CT_max_) for assessing vulnerability to climate change, as it reflects thermal performance over timeframes relevant to ecological processes (Collier *et al*., 2016). This is particularly important under sustained temperature anomalies, such as MHWs, which typically persist for weeks to months – where temperatures exceeding *T*_opt_ lead to a decline in productivity, physiological stress, growth inhibition and potentially mortality if elevated temperatures are prolonged (Collier & Waycott, 2014; Nievola *et al*., 2017).

Accurate assessments of climate change impacts on foundational plant species require a greater understanding of the thermal tolerance both across and within species (i.e. populations). However, recent syntheses on plant thermal tolerances identified a limited number of multi‐species studies, both across species and within a species, and even less information on marine plants (Geange *et al*., 2021; Marba *et al*., 2022). Seagrasses, a group of marine flowering plants, are highly valued for their ecosystem services and contribute to a multitude of environmental and socio‐economic benefits (Orth *et al*., 2006). Despite their recognised importance, seagrasses are among the most threatened ecosystems globally, with an estimated 19%–29% of monitored seagrass area lost since the 1940s (Waycott *et al*., 2009; Dunic *et al*., 2021). In recent decades, MHWs have driven large‐scale mortality events in seagrass meadows, with the impacts often complex and spatially variable (Diaz‐Almela *et al*., 2007; Marbà & Duarte, 2010; Strydom *et al*., 2020). For instance, during the 2010/2011 MHW in Shark Bay, Australia, temperatures rose 2–5°C above summer averages (MHW temperatures: 28–31°C), leading to the loss of *c*. 24% (1310 km^2^) of dense seagrass meadows and the potential release of 2–9 Tg of CO_2_ into the atmosphere over subsequent years (Arias‐Ortiz *et al*., 2018; Strydom *et al*., 2020). However, not all meadows exhibited uniform responses, despite experiencing similar MHW intensities and durations (Strydom *et al*., 2020). There is a critical need for seagrass species‐ and population‐specific assessments of thermal tolerance to better predict which seagrass meadows are most vulnerable to future climate‐driven stressors.

Temperature tolerances among plant species can vary markedly depending on the thermal niche they occupy and their species‐specific adaptations (O'Sullivan *et al*., 2017). In general, tropical species tend to exhibit higher thermal tolerances (or *T*_opt_) than temperate species due to evolutionary processes (Lee *et al*., 2007; Geange *et al*., 2021; Marba *et al*., 2022). For seagrasses, there are relatively few studies that have assessed the *P*–*T* relationship across multiple species (but see Collier *et al*., 2017). However, there is a larger body of work that has examined growth and survival temperature thresholds for seagrasses, with a recent review showing that the upper thermal limit (*T*_limit_) varied by as much as 13°C across 15 species, lowest in temperate species and highest in tropical species (Marba *et al*., 2022). While tropical species may have inherently higher thermal limits, there is some evidence to suggest that temperate species may have a higher plasticity potential due to climatic conditions in temperate zones being more seasonally and spatially variable (Collier *et al*., 2017), which could be advantageous under future projected temperature rises (Barley *et al*., 2021). However, a recent study assessing thermal tolerances for plant germination found that tropical species did not exhibit narrower temperature tolerances compared with temperate species, suggesting that their plasticity potential was similar (Sentinella *et al*., 2020). The study further demonstrated that species growing in tropical zones are more vulnerable to temperature impacts as they are growing closer to their upper thermal limits (Sentinella *et al*., 2020).

Species distributions can extend across a latitudinal temperature gradient, and for some species this gradient may extend across climatic zones (i.e. from temperate to tropical). From the limited work to date, research shows that the thermal tolerance of seagrasses is not always homogeneous across large spatial gradients (100's km), nor is there necessarily a trend of increased tolerance with higher *in situ* temperature (Collier *et al*., 2017; Bennett *et al*., 2022a,b). Furthermore, local‐scale (10′s km) environmental drivers, such as water availability (i.e. drought tolerance), have been shown to alter plant thermal tolerance in terrestrial ecosystems (Curtis *et al*., 2016; Sumner *et al*., 2022), and the same may be true in the seagrasses (e.g. salinity, grazing and tidal zone; Ontoria *et al*., 2020; DuBois *et al*., 2022; Ruggeri *et al*., 2024). Species can tolerate changes in temperature across their distribution at both broad and local scales due to their adaptive capacity, which can influence *T*_opt_ (Bennett *et al*., 2019; Quigley, 2024). For instance, if populations across a species' range display a similar *T*_opt_, this would signify a limited ability to tolerate changes in temperature and warm‐edge populations already growing close to their *T*_opt_ will likely be more vulnerable than the cool‐edge populations. Alternatively, if *T*_opt_ varies across a species' range, regardless of their position (cool‐edge, core or warm‐edge), this could reflect physiological plasticity or local adaptation that enhances photosynthetic performance under local conditions. Therefore, in addition to thermal tolerance data across species, data across an individual species' range are critical for accurately predicting responses to current and future climate conditions, particularly since environmental management decisions are generally made at local to regional scales (Ban *et al*., 2017).

Western Australia (WA) provides an ideal setting for studying seagrass thermal tolerances, with seagrass species spanning broad thermal gradients from temperate to tropical climates (16° latitude). Notably, there are currently no thermal tolerance data available for this region. There is a critical need for this data due to WA's designation as a global hotspot for marine climate impacts – substantial warming at a rate faster than other regions, and more frequent and intense MHW events (Hobday & Pecl, 2014; Kajtar *et al*., 2021). The objective of this study was to identify which species may be vulnerable under future climate scenarios based on physiological thermal tolerance, as well as to provide insight into geographical variation in thermal tolerances which can inform thermal resilience‐building opportunities. Therefore, the aims of this study were to (1) determine the temperature tolerances (*T*_opt_ and CT_max_) of different seagrass species within each of their regions of occurrence (temperate, sub‐tropical and tropical); (2) assess the thermal tolerance among populations of each single species across their thermal latitudinal gradient of occurrence (500–1000 km); and (3) assess the thermal tolerance of a single species across sites at a local scale (*c*. 25km).

## Materials and Methods

### Seagrass species and distribution

**Fig. 1:**
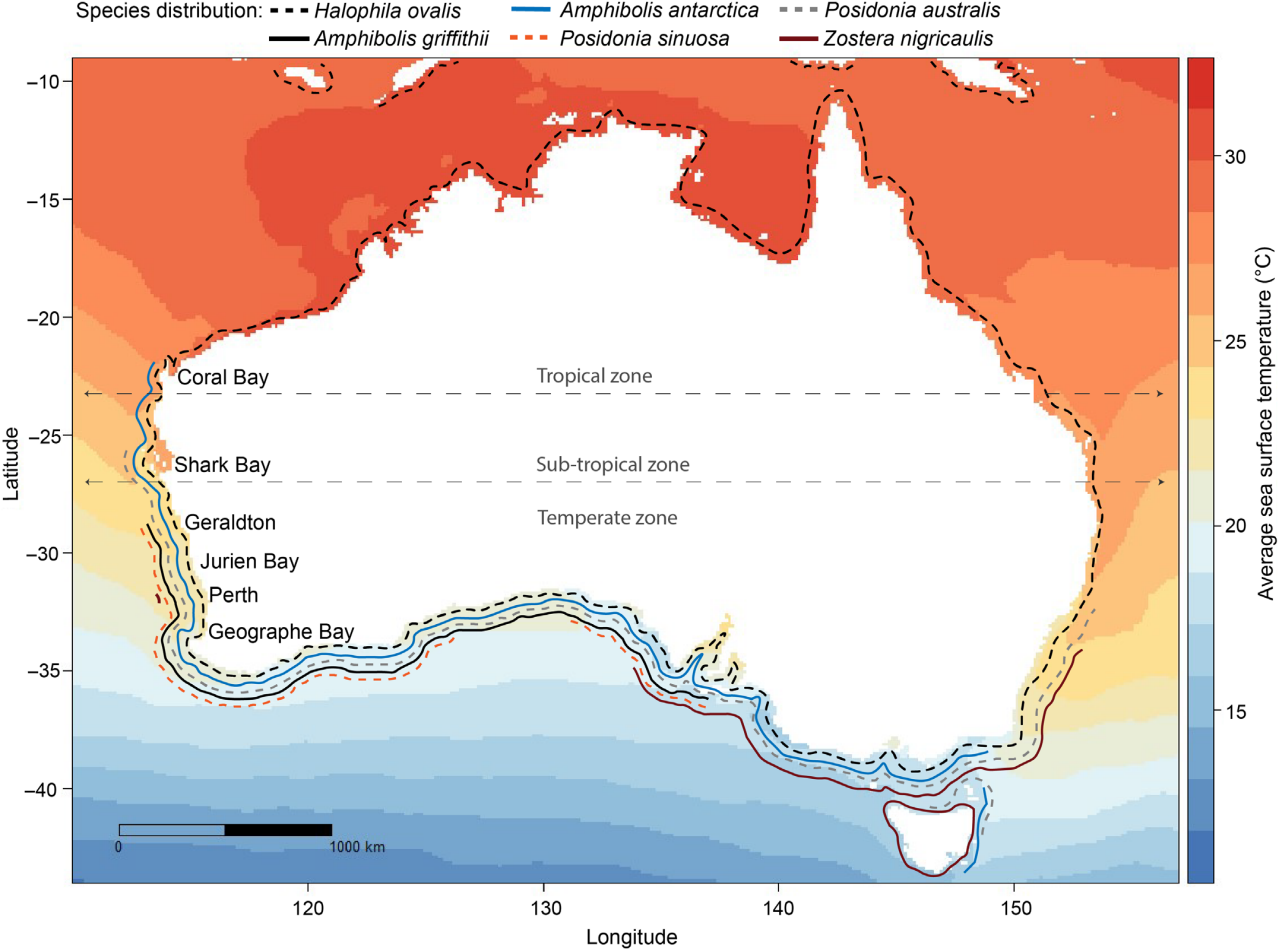
Seagrass species distribution (solid and dashed lines surrounding the coastline) in Australia overlaid with average summer ocean temperatures (blue to red colour scale, based on December to March 10 yr average from 2011 to 2022; temperature data sourced from NOAA satellite imagery). Six locations were assessed in this study: Coral Bay as tropical, Shark Bay as sub‐tropical and Geraldton, Jurien Bay, Perth and Geographe Bay as temperate.

**Fig. 2:**
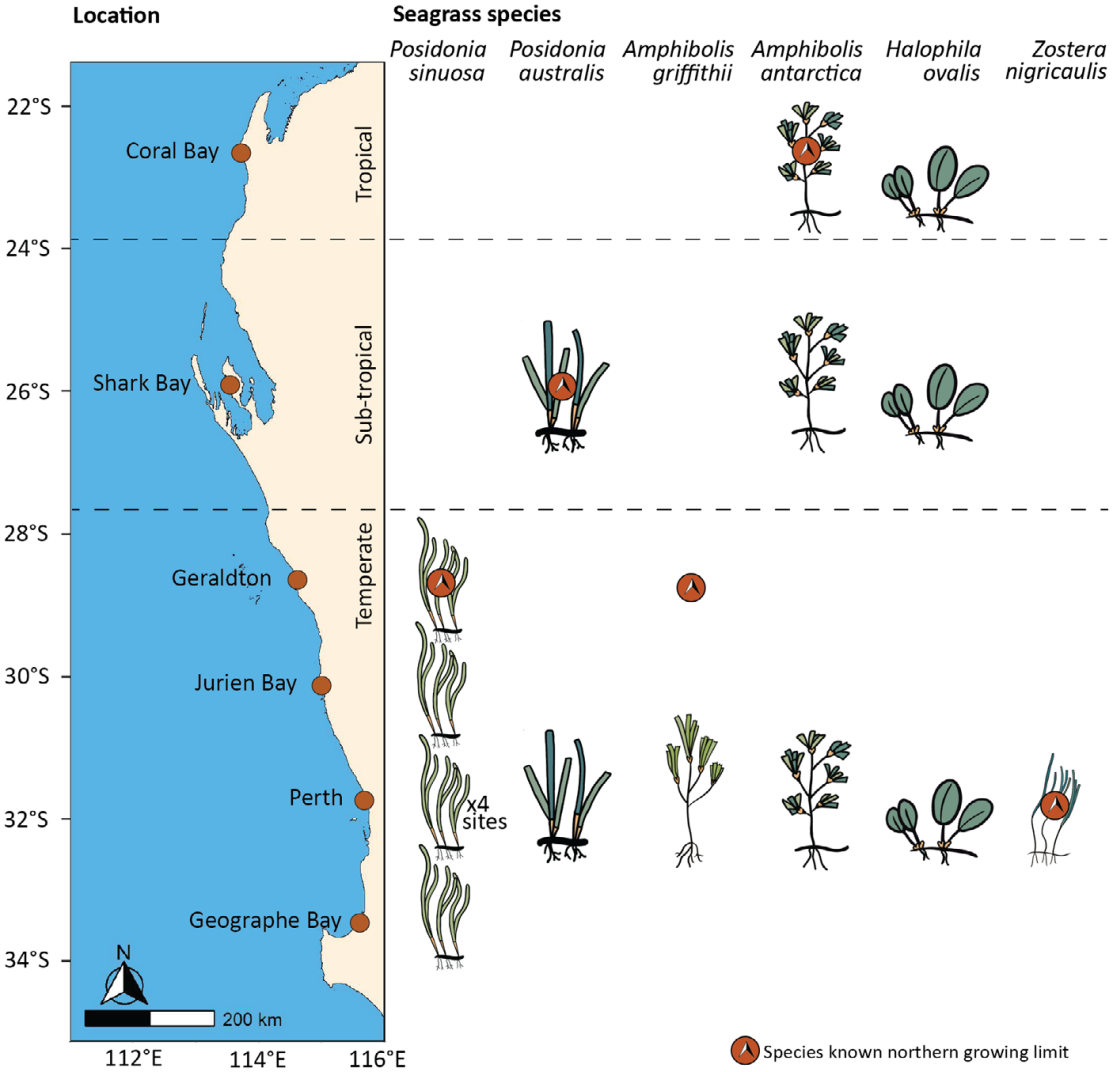
Visual representation of seagrass species collected at each location across a latitudinal gradient (23°S–33°S latitude; *c*. 1000 km). The most northern growing limit of each species represents the warmest region in which they occur; however, *Amphibolis griffithii* was not sampled from its northernmost population (Geraldton). Across species, comparisons can be made in temperate Perth for all six species, sub‐tropical Shark Bay for three species (*Posidonia australis*, *Amphibolis antarctica* and *Halophila ovalis*) and tropical Coral Bay for two species (*A. antarctica* and *H. ovalis*). Population‐level comparisons can be made for *Posidonia sinuosa* (temperate region), *P. australis* (temperate, sub‐tropical), *A. antarctica* (temperate, sub‐tropical and tropical) and *H. ovalis* (temperate, sub‐tropical and tropical). At the local scale, *P. sinuosa* was also sampled at four sites within the Perth region.

We assessed the thermal tolerance of five temperate seagrass species (*Posidonia sinuosa Cambridge & J.Kuo*, *Posidonia australis Hook.f.*, *Amphibolis antarctica (Labill.) Asch.*, *Amphibolis griffithii* (J.M.Black) Hartog, and *Zostera nigricaulis (Syn. Heterozostera nigricaulis J.Kuo)*), and one globally distributed species, *Halophila ovalis (R.Br.) Hook.f*. The geographic range for some of the temperate species (*P. australis* and *A. antarctica*) extends into sub‐tropical and tropical climatic zones (Fig. 1). For this study, we defined tropical waters as north of *c*. 23.5°S (the Tropic of Capricorn), sub‐tropical as *c*. 23.5–28°S and temperate as south of *c*. 28°S. All of these species were assessed in Perth coastal waters (temperate zone), and four of these six species were assessed at two to four locations over a latitudinal gradient (Fig. 2; Table 1). Additionally, *P. sinuosa* was collected from four sites in the Perth location to assess local scale variability (Table 1). We define the locations as tropical (Coral Bay), sub‐tropical (Shark Bay) and temperate (Geraldton, Jurien Bay, Perth and Geographe Bay; Fig. 1).

**Table 1:** Seagrass species, collection location and site with corresponding GPS coordinates (latitude and longitude), and environmental data.

Species	Collection location	Collection site^1^	Latitude	Longitude	Location water temp range (°C)^2^	Water temp at collection (°C)	Salinity at collection (ppt)	Water depth (m)^5^
*Posidonia sinuosa*	Geraldton		28.748°S	114.610°E	17–24	21	33.4	8
	Jurien Bay		30.330°S	115.031°E	17–24	19	33.6	4
	Perth	Shoalwater	32.272°S	115.690°E	15–23	18	33.6	2
	Perth	Cockburn 1	32.241°S	115.707°E	15–23	21	35.1	4
	Perth	Cockburn 2	32.157°S	115.684°E	15–23	21	35.1	4
	Perth	Cockburn 3	32.129°S	115.738°E	15–23	21	35.1	3
	Geographe		33.616°S	115.128°E	15–23	15	35–36^4^	4
*Posidonia australis*	Shark Bay		25.824°S	113.463°E	17–26	17.5	39.5	2
	Perth		32.136°S	115.746°E	15–23	16	33.6	2
*Amphibolis antarctica*	Coral Bay		22.723°S	113.710°E	21–26	19^3^	34.0	8
	Shark Bay		25.824°S	113.463°E	17–26	17.5	39.5	2
	Perth		32.272°S	115.690°E	15–23	18	33.6	2
*Amphibolis griffithii*	Perth		32.272°S	115.690°E	15–23	18	33.6	2
*Halophila ovalis*	Coral Bay		23.168°S	113.762°E	21–26	21	34.0	2
	Shark Bay		26.031°S	113.554°E	17–26	25	41.5	1.5
	Perth		32.272°S	115.690°E	15–23	15	33.6	2
*Zostera nigricaulis*	Perth		32.136°S	115.746°E	15–23	17	33.6	2

Environmental variables include the 10‐yr average water temperature range for each location (summer and winter), water temperature and salinity at the time of plant collection and water depth. For *P. sinuosa* in the Perth region, individual collection sites are listed separately, as multiple sites were assessed. Collection time (month yr^−1^) is provided in Supporting Information Table S1.

^1^ Cockburn (Cockburn Sound) *P. sinuosa* local meadow names: (1) Southern Flats; (2) Garden Island; (3) Woodman.

^2^ Water temperature data 10‐yr average from NOAA Coral Reef Watch Daily Global 5 km Satellite Data, corrected with benthic logger data provided by DBCA, Mid‐West Ports and Busselton Jetty. See ‘Temperature data’ in the Materials and Methods section for further details.

^3^ Note water temperature at the time of collection is lower than the average minimum water temperature range. This is likely due to cooler water temperatures at the substrate in 8 m of water.

^4^ Salinity not collected at site for Geographe Bay location, range provided from Port Geographe Bay Water Quality Report (O2 Marine, 2021).

^5^ Water depth at time of collection.

The seagrass species selected can be classified as colonising, opportunistic and persistent, following the life‐history strategy framework outlined in Kilminster *et al*. (2015), which relates species traits to their ability to resist and recover from pressures. Smaller, faster growing colonising genera (e.g. *Halophila*) have a low physiological resistance (duration) to pressures (e.g. MHWs); however, they have the ability to rapidly recover. Comparatively, the larger, persistent genera (e.g. *Posidonia*) have a greater ability to withstand pressures but are slower to recover. Opportunistic genera (e.g. *Zostera*) are somewhere in between colonising and persistent and combine elements of both resistance and recovery strategies.

### Plant collection and acclimation

Plant samples were collected from sub‐tidal seagrass meadows to assess whether the *P*–*T* relationship varied: (1) among species, with two to six species collected from each climatic zone; (2) across a latitudinal gradient (500–1000 km) for four species; and (3) within a single location (four sites) for one species, *P. sinuosa* (Fig. 2). The time of year when plants were collected typically varied (Supporting Information Table S1); however, previous research has shown that *P*–*T* metrics, such as *T*_opt_ and CT_max_, do not significantly vary across seasons (Collier *et al*., 2017). For each site and species, *c*. 40 whole plants were collected, with plant size varying depending on species and growth form. To account for the whole‐plant carbon budget – including both photosynthesis and respiration, which can influence the *P*–*T* relationship (Collier *et al*., 2017) – intact plants were collected by gently fanning away sediment and carefully digging beneath the roots and rhizome. Plants were then placed in a seawater‐filled cooler box with aeration for transport to the laboratory. At each collection time, temperature and salinity were recorded to determine initial laboratory acclimation conditions (Table 1).

In the laboratory, plants were gently cleaned to remove epiphytes and then planted into 50‐l aquaria containing *c*. 10 cm layer of unsorted, washed quartz river sand. Each aquarium was connected to a 30‐l sump tank via a pump to maintain water flow. Water temperature (regulated with heaters) and salinity mimicked field collection conditions (Table 1). Each species–location combination was housed in its own dedicated tank, and plants were tested sequentially after collection to maintain plant health. Plants were provided with *c*. 180 μmol photons m^−2^ s^−1^ (above approximating saturating irradiance; Lee *et al*., 2007) using marine aquarium light‐emitting diode modules with a full spectrum light (MarinTech™ Pty Ltd) on a 12 h : 12 h, light : dark cycle. Light intensity at the base of the canopy was measured using a micro‐PAR sensor (In‐Situ Marine Optics™). Plants were left for 2 d to acclimate after which dark‐adapted maximum quantum yields were taken on five separate plants to assess their acclimation. Plants were not used for experimentation until they were considered healthy; if they had a dark‐adapted yield of 0.7–0.75 (Ralph & Burchett, 1995).

### Photosynthesis–temperature determinations

For each seagrass species and location (or site) combination, laboratory measurements were conducted to assess seagrass productivity (NP_max_), optimum temperature (*T*_opt_) at which maximum photosynthesis occurred, critical thermal maximum temperature (CT_max_) where photosynthesis is zero, respiration (*R*) and metabolic rate change (*Q*_10_). Seagrass photosynthesis and respiration were quantified via oxygen production or consumption. For each species–location combination, one temperature‐controlled water bath was used, with five replicate incubation chambers, each containing three to five whole plants (depending on species) of similar size and a sixth control chamber with no plant material to account for any background oxygen flux. Multiple whole plants (four to five leaf pairs with an apical meristem for *H. ovalis* and more than three shoots/stems for other species) were incubated in sealed, transparent cylindrical acrylic chambers, with the size (15–55 cm length) and orientation (vertical or horizontal to mimic natural plant orientation) of chambers adjusted depending on the species being assessed (Table S2). Water within the chambers was circulated using small submersible pumps (flow rate 360 l h^−1^). Dissolved oxygen concentrations within the chambers were measured using FireSting™ 3 mm robust REDFLASH technology sensors (PyroScience, Aachen, Germany) inserted through the chamber wall and connected through a four‐channel metre to a computer recording mg of O_2_ (See Fig. S1 for experimental set‐up). Oxygen electrodes were calibrated using the manufacturer's two‐point method (0% and 100% air saturated water).

To maintain a stable temperature (±0.25°C), incubation chambers were submerged in a tank with 200–450 l of seawater, which was circulated through a heater‐chiller unit set to the appropriate experimental temperature. Temperatures ranged from 15°C to 42°C, where cooler temperatures were reached using the chiller function, and warmer temperatures were reached using the heater function as well as additional titanium heaters to elevate to the higher temperatures. While temperature control of the bath was precise to within ±0.25°C, internal chamber temperatures were also measured using a submersible temperature sensor (accuracy ±0.5°C) connected to the FireSting O_2_ metre, and this was the temperature value used for all experimental recordings. Consequently, thermal tolerance parameters (*T*_opt_ and *T*_max_) were reported to the nearest 0.5°C to reflect sensor accuracy. For each temperature treatment, oxygen concentrations (measured every second) were first measured in the dark for seagrass respiration (*R*), and then, the plants were illuminated by full spectrum lights (MarinTech™ Pty Ltd) above a saturating light level of 400 μmol m^−2^ s^−1^ to measure combined photosynthesis and respiration (i.e. representative of whole‐plant net photosynthesis (NP)). This was repeated at 8–10 temperature steps in 3°C increments (15°C, 18°C, 21°C, 24°C, 27°C, 30°C, 33°C, 36°C, 39°C and 42°C), with the number of temperature steps varying depending on species: 8 for *Z. nigricaulis*, 9 for all other temperate species and 10 for *H. ovalis*. Temperature steps were selected to capture the full photosynthetic response range, with experiments terminated when live oxygen data showed negative oxygen consumption under saturating light – allowing for accurate estimation of CT_max_.

Experiment duration ranged from 8 to 12 h, with length dependent on the number of temperature steps. The time to reach each experimental temperature averaged 45 ± 8 min, consistent with previous chamber studies that calculated *T*_opt_ for seagrasses (Pedersen *et al*., 2016). Note that the ecological relevance of short‐term experiments in assessing seagrass vulnerability to climate impacts is discussed in ‘Thermal vulnerability assessment across and within species for current and future climate scenarios’ in the Discussion. Once the chamber temperature stabilised, plants were incubated in the dark for 12 min to measure respiration. Plants were then illuminated, and a 5‐min stabilisation period was allowed to enable the physiological adjustment from respiration to photosynthesis (extended up to 10 min if required for a stable oxygen slope). Following this, plants remained under the light for a further 10 min to measure NP. Where necessary, seawater within the chambers was refreshed in between temperature steps if the oxygen level fell below 2 mg O_2_ l^−1^ to prevent hypoxia. At the end of the experiment, data were extracted for both dark and light steps at each temperature, retaining a 10‐min portion of stable oxygen data for each, with the initial 2 min of each step discarded (i.e. 10 min for dark and 10 min for light at each temperature). Plants from each replicate chamber were retained and separated into aboveground and belowground tissue. All plant material was dried at 60°C for 48 h and then weighed to derive biomass in g per dry weight (DW) to standardise oxygen measurements.

### Photosynthesis–temperature curve fitting

For each replicate incubation (and control) and for all temperatures, oxygen concentration was plotted against time, where the relationship between the two parameters (*R* ^2^) was ≥ 0.9. Rates of oxygen exchange were normalised to mg O_2_ g^−1^ DW^−1^ h^−1^. Oxygen concentrations in the control chamber (without seagrass) were monitored throughout each experiment, to detect if there was no significant change in oxygen concentration over time. To minimise background microbial activity, the experimental tank was cleaned and fresh seawater was used for each experiment.

Photosynthetic rates were expected to follow the general trend of a gradual increase up to *T*_opt_, followed by a steep decline beyond this point towards CT_max_ (Adams *et al*., 2017), and respiration was expected to show an increase with increasing temperature. For each incubation, *P*–*T* data were fitted to the Yan and Hunt model (Yan & Hunt, 1999) to obtain maximum metabolic rates and thermal tolerances for photosynthesis. The Yan and Hunt model is most appropriate for this type of data (Adams *et al*., 2017; Collier *et al*., 2017) to estimate thermal optima (*T*_opt_), critical thermal maxima (CT_max_) and maximum net photosynthesis (NP_max_) at *T*_opt_.

(Eqn 1) $$\mathrm{NP}\left(T\right)={\mathrm{NP}}_{\max}\left(\frac{{\mathrm{CT}}_{\max}-T}{{\mathrm{CT}}_{\max}-T_{\mathrm{opt}}}\right){\left(\frac{T}{T_{\mathrm{opt}}}\right)}^{T_{\mathrm{opt}}/\left({\mathrm{CT}}_{\max}-T_{\mathrm{opt}}\right)}$$

**Fig. 3:**
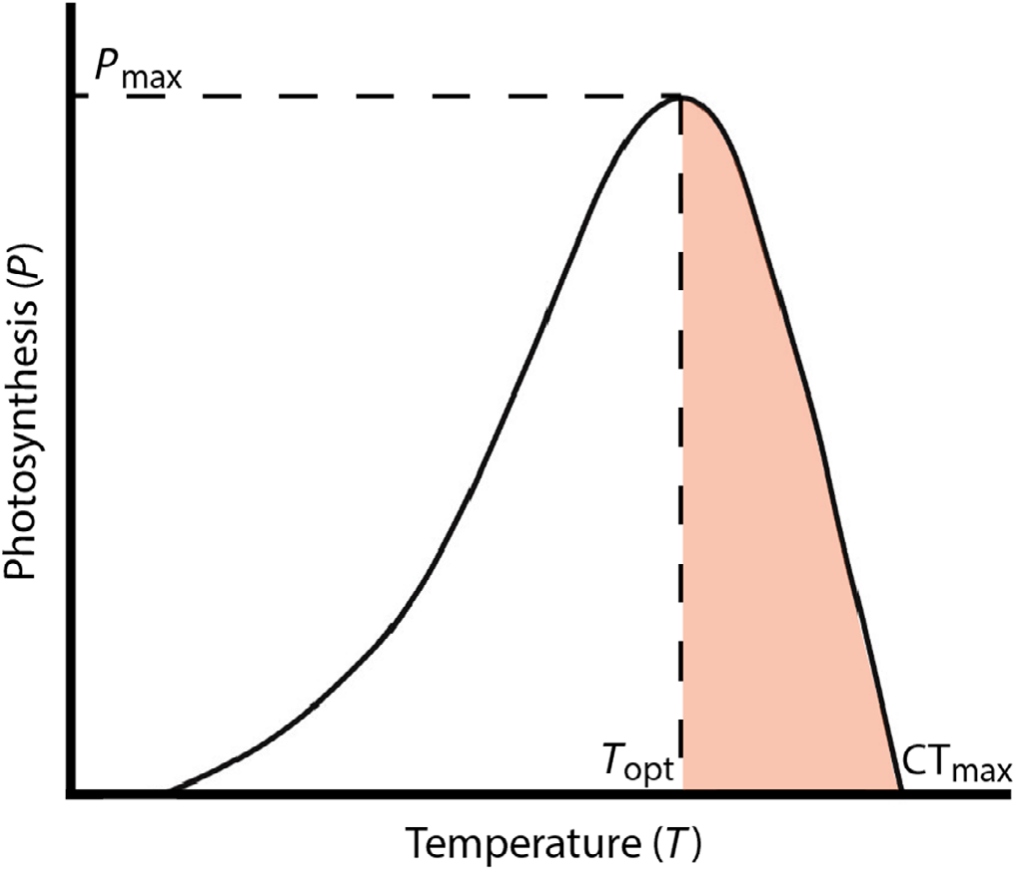
Illustration of a typical photosynthesis–temperature (*P*–*T*) performance curve. The photosynthetic rate increases with temperature until reaching a maximum photosynthesis (*P*_max_), which can be expressed as either net (NP_max_) or gross (GP_max_) photosynthesis. The thermal optimum (*T*_opt_) corresponds to *P*_max_, beyond which photosynthesis declines sharply towards the critical thermal maximum (CT_max_) where photosynthesis reaches zero. For seagrass, the orange shaded region above *T*_opt_ for photosynthesis represents elevated vulnerability to sustained high temperatures, and potential mortality if temperature stress is prolonged.

In this equation, NP(*T*) is the biological rate of NP at temperature *T* (°C), which represents NP (mg O_2_ g DW^−1^ h^−1^), NP_max_ is the maximum rate (mg O_2_ g DW^−1^ h^−1^) which occurs at the optimum temperature *T*_opt_ (°C), and CT_max_ (°C) is the temperature greater than the optimum at which photosynthesis drops to zero (Fig. 3; Collier *et al*., 2017). Using nonlinear regression (MATLAB Statistics and Machine Learning Toolbox R2022a), Eqn (1) was fitted to seagrass NP of whole plants under saturating irradiance (which takes both leaf respiration and belowground respiration into consideration), and gross photosynthesis (GP) for the six species, location and site combinations. GP data were obtained by subtracting R data from NP data. NP data are presented in ‘Thermal performance between different species’, ‘Thermal performance across latitudinal gradients and climate zones’ and ‘Thermal performance at the local scale (within a location)’ in the Results section; GP data are presented in Table S3.

Below the optimum temperature, the *Q*_10_ coefficient is used to predict the rate of metabolic change, which is the factor by which there is an increase in the biological rate due to a temperature increase of 10°C (Atkin & Tjoelker, 2003). Following methods from Collier *et al*. (2017), a second model was fitted to estimate *Q*_10_ values by fitting the temperature dependence of seagrass NP and respiration (*R*) for all species, location and site combinations. Based on the *P*–*T* model (Eqn 1), only data that were predicted to fall below *T*_opt_ were fitted to Eqn 2. The second model was an exponential function of the form.

(Eqn 2) $$\mathrm{NP}\left(T\right)={\mathrm{NP}}_{0}Q_{10}^{\left(T-T_{0}\right)/10}$$

where NP_0_ (mg O_2_ g DW^−1^ h^−1^) is the biological rate at a reference temperature *T*_0_ (°C). We set *T*_0_ = 20°C, following the protocol of (Baird *et al*., 2016).

### Scaling up physiological thermal estimates using daily plant metabolism

To explore the effect of the climatic zone and seasonality on daily net metabolism, we also used the raw data for instantaneous NP(*T*) and *R*(*T*) to compute approximate daily net photosynthesis data, NP_daily_(*T*), for three different day‐night cycles. These computed NP_daily_(*T*) data were then fitted to the Yan and Hunt model (Eqn 1).

More specifically, to estimate the productivity for daily plant metabolism (mg O_2_ g DW^−1^ d^−1^) the total daily light was calculated for each climatic zone (tropical, sub‐tropical and temperate) over both winter and summer periods (daylight hours extracted from https://www.timeanddate.com/). As there are different light regimes over both climatic zones and seasons, the ratio of NP : R varies, and therefore, the shape of the curve and thermal tolerance parameters (*T*_opt_ and *T*_max_) have the potential to change. The following equations integrate location‐specific and season‐specific light regimes by utilising NP for daylight hours and R for nondaylight hours.

(Eqn 3) $$\begin{array}{l} \text{Assumed for summer in}\;\mathrm{all}\;\text{climatic zones}\;:\;\\ \;{\mathrm{NP}}_{\text{daily}}\left(T\right)\;\approx \;14\times \mathrm{NP}\left(T\right)+10\times R\left(T\right) \end{array}$$

(Eqn 4) $$\begin{array}{l} \text{Assumed for winter in temperate zone}\;:\;\\ \;{\mathrm{NP}}_{\text{daily}}\left(T\right)\;\approx \;10\times \mathrm{NP}\left(T\right)+14\times R\left(T\right) \end{array}$$

(Eqn 5) $$\begin{array}{l} \text{Assumed for winter in tropical and}\;\mathrm{sub}‐\text{tropical zones}\;:\;\\ \;{\mathrm{NP}}_{\text{daily}}\left(T\right)\;\approx \;11\times \mathrm{NP}\left(T\right)+13\times R\left(T\right) \end{array}$$

In Eqns (Eqn 3), (Eqn 4), (Eqn 5), NP(*T*) is the instantaneous biological rate of NP at *T* (°C), which represents NP (mg O_2_ g DW^−1^ h^−1^), *R*(*T*) is the instantaneous biological rate of respiration (*R*) at *T* (°C), which represents respiration (*R*; mg O_2_ g DW^−1^ h^−1^), and NP_daily_(*T*) is the daily biological rate of NP incorporating the 24 h day‐night cycle (mg O_2_ g DW^−1^ d^−1^). In Eqn 3, daily plant metabolism rates were calculated for summer based on daylight hours for all locations (14 h : 10 h, light : dark). For winter, daylight hours change across climatic zones, and therefore, for temperate locations (Eqn 4), 10 h : 14 h, light : dark, and for tropical locations (Eqn 5), 11 h : 13 h, light : dark. These data were then fitted to Eqn 1, and data are presented in Table S4. For some data in winter, the model could not be fitted as NP at lower temperatures (15–21°C) had negative values (within normal temperature ranges, the Yan and Hunt model cannot predict negative values). In these instances, we have reported data as not available (NA).

### Temperature data

Satellite sea surface temperature (SST) data were extracted from the NOAA CoastWatch ERDDAP data server (dataset ID: noaacrwSSTanomalyDaily) at a spatial resolution of 5 km grid cells for each study location where the plants were collected as per Table 1. Average summer temperatures were calculated as a 10‐yr mean (2013–2022) across December, January, February and March. Following Baldock *et al*. (2014), SST data were corrected using *in situ* benthic temperature logger data from each location to account for surface‐to‐benthic thermal discrepancies. Maximum temperatures during the well‐documented 2010/2011 MHW in WA (Pearce & Feng, 2013) were also extracted for each location (Table 1) from NOAA and corrected using the same methodology. All temperature data were rounded to the nearest 0.5°C.

### Thermal vulnerability assessment

To be able to put conservation efforts into place under climate change, we need to understand which species and populations (locations and sites) are vulnerable to temperature impacts. We have used the thermal tolerance information (*T*_opt_ hourly rates; based on methods in ‘Photosynthesis–temperature curve fitting’ in the Materials and Methods section) generated in this study to assess seagrass vulnerability to current conditions as well as future ocean warming and MHWs. *T*_opt_ denotes the temperature at NP_max_, and therefore, temperatures greater than *T*_opt_ will result in negative declines in productivity. We interpret temperatures > *T*_opt_ as the temperature threshold to which species would likely be more vulnerable to ocean warming or MHWs. The three temperature scenarios are as follows: (1) The current summer ambient temperature for that location; (2) The 2010/2011 MHW temperature from a given location; and (3) A future warming scenario of an increase in 2.2°C above current summer conditions (2.2°C is based on an average effort to curb emissions globally; Representative Concentration Pathway (RCP) scenario 6.0 in 2100; IPCC, 2023). When comparing methods for extracting *T*_opt_ from hourly instantaneous rates (‘Photosynthesis–temperature curve fitting’ in the Materials and Methods section) to daily plant metabolism rates (‘Scaling up physiological thermal estimates using daily plant metabolism’ in the Materials and Methods section), we found a maximum *T*_opt_ difference of 2°C (range 0–2°C; Table S5) in summer, and therefore, we have used the variability of 2°C as a buffer around concern ratings. A ‘concern’ rating for vulnerability was defined as Low, where water temperature of either scenario was equal to or greater than 2°C below *T*_opt_; Moderate, where water temperature was less than 2°C (0–1.99°C) below *T*_opt_; or High, where the water temperature was above *T*_opt_.

## Results

### Thermal performance

**Fig. 4:**
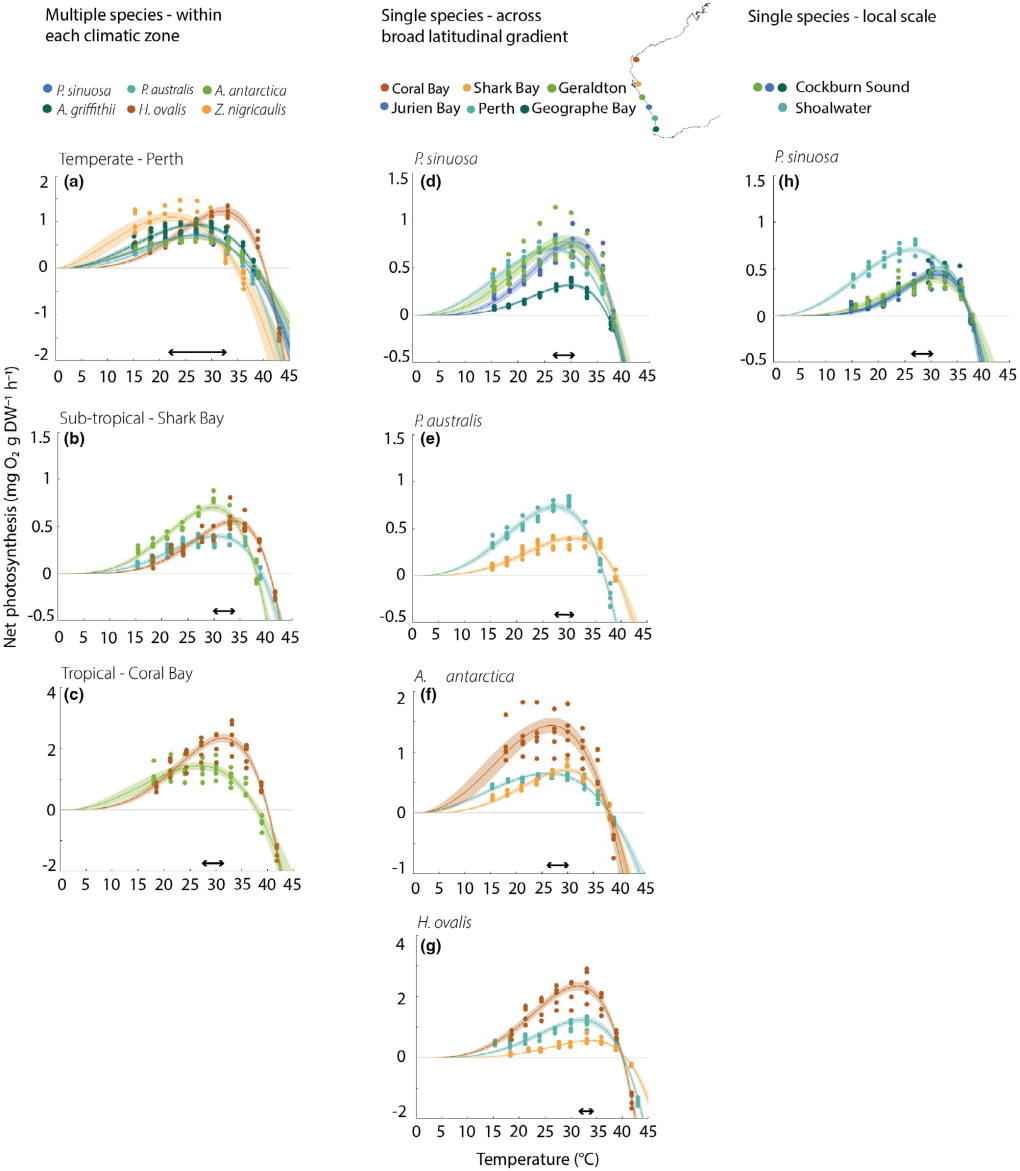
Net photosynthesis–temperature curves at temperatures ranging from 15°C to 42°C: for multiple seagrass species within each climatic zone (a) temperate Perth, (b) sub‐tropical Shark Bay and (c) tropical Coral Bay; single species across broad latitudinal scales (d) *Posidonia sinuosa*, (e) *Posidonia australis*, (f) *Amphibolis antarctica* and (g) *H. ovalis*; and for a single species across a local scale (h) *P. sinuosa* at four sites in Perth location. [Correction added on 19 January 2026, after first online publication: the temperature range in the preceding sentence has been corrected.] The double‐ended arrows indicate the range in which *T*_opt_ varied for a given species, location or site combination. (*n* = 5). Modelled fits are shown by the coloured lines, with the shading representative of the 95% CI in the model fits. Note the different scales on the *Y*‐axes.

All species followed a temperature‐dependent relationship, showing a gradual increase in photosynthesis with temperature to *T*_opt_, generally followed by a rapid decline to CT_max_ (Fig. 4). For all species, respiration measured in the dark increased with temperature and was generally highest at the highest temperature tested (Table 2).

**Table 2:** Data are based on net hourly photosynthesis–temperature curves (mg O_2_ g DW^−1^ h^−1^): *T*_opt_, CT_max_, *Q*_10_ and maximum net productivity (NP_max_) at *T*_opt_ values.

Species	Location	Site^1^	Temperature (°C)			Net photosynthesis			
			Summer average	Summer average + 2.2	2010/2011 marine heatwave	*T*_opt_ (°C)	CT_max_ (°C)	*Q*_10_	NP_max_, at *T*_opt_ (mg O_2_ g DW^−1^ h^−1^)
*Posidonia sinuosa*	Geraldton		24	26.2	28	28.5 ± 0.5	38.0 ± 0.3	2.0 ± 0.3	0.78 ± 0.04
	Jurien Bay		24	26.2	29	31.0 ± 0.3	38.0 ± 0.2	2.2 ± 0.2	0.77 ± 0.03
	Perth	Shoalwater	23	25.2	26	26.5 ± 0.3	37.5 ± 0.1	1.9 ± 0.2	0.70 ± 0.01
	Perth	Cockburn 1	23	25.2	26	31.0 ± 0.3	37.5 ± 0.5	2.9 ± 0.3	0.44 ± 0.02
	Perth	Cockburn 2	23	25.2	26	31.0 ± 0.3	37.5 ± 0.2	3.1 ± 0.4	0.48 ± 0.03
	Perth	Cockburn 3	23	25.2	26	30.0 ± 0.4	38.0 ± 0.3	2.1 ± 0.3	0.41 ± 0.02
	Geographe		23	25.2	26	30.0 ± 0.3	37.0 ± 0.1	3.3 ± 0.2	0.32 ± 0.01
*Posidonia australis*	Shark Bay		26	28.2	31	30.5 ± 0.3	39.0 ± 0.1	1.9 ± 0.2	0.39 ± 0.01
	Perth		23	25.2	26	27.5 ± 0.3	37.0 ± 0.1	1.8 ± 0.1	0.73 ± 0.02
*Amphibolis antarctica*	Coral Bay		26	28.2	29	27.0 ± 0.7	38.0 ± 0.3	1.3 ± 0.3	1.44 ± 0.06
	Shark Bay		26	28.2	31	29.5 ± 0.3	38.0 ± 0.2	2.2 ± 0.1	0.70 ± 0.02
	Perth		23	25.2	26	26.0 ± 0.4	38.0 ± 0.2	1.4 ± 0.1	0.65 ± 0.01
*Amphibolis griffithii*	Perth		23	25.2	26	27.0 ± 0.4	38.5 ± 0.2	1.6 ± 0.2	0.94 ± 0.02
*Halophila ovalis*	Coral Bay		26	28.2	29	31.5 ± 0.3	40.0 ± 0.1	2.0 ± 0.2	2.34 ± 0.07
	Shark Bay		26	28.2	31	33.5 ± 0.3	40.5 ± 0.1	2.4 ± 0.2	0.56 ± 0.02
	Perth		23	25.2	26	32.0 ± 0.4	40.0 ± 0.2	1.8 ± 0.1	1.23 ± 0.05
*Zostera nigricaulis*	Perth		23	25.2	26	22.5 ± 0.9	35.0 ± 0.4	1.1 ± 0.3	1.11 ± 0.05

Average summer temperature, ocean warming (average summer +2.2°C; RCP 6.0), and 2010/2011 marine heatwave data for each species, location combination, where for each scenario blue is low concern (≥ 2°C below *T*_opt_), yellow is moderate concern (= within 0–1.99°C of *T*_opt_), and orange is high concern (= above *T*_opt_). Respiration, gross photosynthesis and AG : BG values are in Supporting Information Table S3. Values are presented as mean ± standard error (SE). *T*_opt_ and CT_max_ are reported to the nearest 0.5°C.

^1^ Cockburn (Cockburn Sound) populations: (1) Southern Flats; (2) Garden Island; (3) Woodman.

Overall, based on NP data (mg O_2_ g DW^−1^ h^−1^) there was a larger variation among species for *T*_opt_ (1–10°C) and CT_max_ (0–5°C), than across locations for the same species (*T*_opt_ 0–4°C, CT_max_ 0–2°C), and sites within a location for the same species (*T*_opt_ 0–4°C and CT_max_ 0°C). *T*_opt_ for daily plant metabolism (mg O_2_ g DW^−1^ d^−1^) varied from 0°C to 2°C from hourly rates in summer (mg O_2_ g DW^−1^ h^−1^; Table S5). In general, there were no substantial differences in the *Q*_10_ coefficient among species, across locations or sites within location (Table 2; Fig. S2).

#### Thermal performance between different species

In the temperate location of Perth where all six species were assessed, there was a clear difference in *T*_opt_ among species, varying by almost 10°C (Table 2; Fig. 4a). For the larger species in Perth (*P. australis*, *A. antarctica* and *A. griffithii*), *T*_opt_ and CT_max_ varied by *c*. 1°C, ranging from 26°C to 27°C for *T*_opt_ and 38°C to 39°C for CT_max_ (see ‘Thermal performance at the local scale (within a location)’ in the Results section for *P. sinuosa* in Perth). *Zostera nigricaulis* had the lowest *T*_opt_ (22.5°C) and CT_max_ (35°C). *Halophila ovalis*, a globally distributed species, had the highest *T*_opt_ of 32°C and highest CT_max_ of 40°C.

In the sub‐tropical location (Shark Bay), *A. antarctica* and *P. australis T*_opt_ differed by 1°C; 29.5°C; and 30.5°C, respectively (Fig. 4b). CT_max_ was 38°C for both species. *Halophila ovalis* had a higher *T*_opt_ and CT_max_, of 33.5°C and 40.5°C, respectively.

In the tropical location (Coral Bay), *T*_opt_ varied by *c*. 4°C, with *H. ovalis* having a higher *T*_opt_ than *A. antarctica*, at 31.5°C and 27°C, respectively (Fig. 4c). CT_max_ across the two species had a lower variation (than *T*_opt_), varying by *c*. 2°C, with *H. ovalis* having a higher CT_max_ than *A. antarctica*, at 40°C and 38°C, respectively.

#### Thermal performance across latitudinal gradients and climate zones

Thermal optima varied across locations for a single species by *c*. 2.5–4°C, but the amount of variation was species‐dependent (Table 2; Fig. 4d–g). The variation in *T*_opt_ showed no consistent trend across climatic zones (temperate, sub‐tropical and tropical).

The *T*_opt_ for *P. sinuosa* varied by *c*. 4°C across the four temperate locations (Geraldton, Jurien Bay, Perth and Geographe Bay) spanning *c*. 500 km and 5° latitude. There was no consistent pattern in *T*_opt_ along the latitudinal gradient (Table 2; Fig. 4d). Geographe Bay, the population growing in a location with cooler waters, had a similar *T*_opt_ to Jurien Bay (30°C and 31°C, respectively), one of the warmer locations, and the Geraldton population (most northern) had a 2°C lower *T*_opt_ (29°C) than Jurien Bay. The Perth location is discussed in ‘Thermal performance at the local scale (within a location)’ in the Results section and depending on the site had a similar *T*_opt_ to Geographe Bay and Jurien Bay; however, one of the sites also had the lowest *T*_opt_ (26.5°C; Table 2). By contrast, there was a trend of increased CT_max_ from Geographe Bay, 37°C, to Geraldton, 38°C. The rate of *Q*_10_ at most locations was not substantially different (1.9–2.2), except for Geographe Bay where plants had a *Q*_10_ of 3.3 (Table 2; Fig. S2).

*Posidonia australis* was assessed in temperate Perth and sub‐tropical Shark Bay, *c*. 700 km and 7° latitude apart. Both *T*_opt_ and CT_max_ were higher at the sub‐tropical location, compared with the temperate location (Fig. 4e). *T*_opt_ varied by *c*. 4°C, 31°C in the sub‐tropical location and 27°C in the temperate location and CT_max_ varied by *c*. 2°C (39°C and 37°C, respectively).

*Amphibolis antarctica* was assessed across three climatic zones: temperate (Perth), sub‐tropical (Shark Bay) and tropical (Coral Bay), spanning *c*. 1000 km and 10° latitude (Fig. 4f). There was no clear trend for *T*_opt_ along the latitudinal gradient. *T*_opt_ varied by *c*. 3.5°C, lowest in the temperate location (26°C) and highest at the sub‐tropical location (29.5°C), with the tropical location *T*_opt_ (27°C) being more similar to the temperate location. *A. antarctica* had a CT_max_ of 38°C for all locations.

*Halophila ovalis* showed the least variation in *T*_opt_ across locations, varying by 1–2°C (Fig. 4g). The sub‐tropical location had the highest *T*_opt_ of 33.5°C. The temperate location (Perth) had a slightly higher *T*_opt_ than the tropical location (Coral Bay), 32°C and 31.5°C, respectively. CT_max_ for *H. ovalis* ranged from 40°C to 40.5°C across all locations tested.

#### Thermal performance at the local scale (within a location)

Thermal optima varied by *c*. 1–4°C across sites (*c*. 25 km range) for *P. sinuosa* in the Perth location (Table 2; Fig. 4h). *Posidonia sinuosa* meadows within Cockburn Sound (Southern Flats, Garden Island, Woodman) all had a similar *T*_opt_, ranging from 30°C to 31°C. Shoalwater (within Warnbro Sound) had a lower *T*_opt_ of 26.5°C. The CT_max_ was similar among sites, 37.5–38°C.

### Vulnerability assessment

Here, we assessed seagrass vulnerability (species/population combinations) to three temperature scenarios using *T*_opt_. When assessing *T*_opt_ against location‐specific average summer ambient temperatures alone, almost all species and location combinations could be attributed to a rating of low concern, except for *Z. nigricaulis* (Table 3). Similarly, under the ocean warming scenario (summer ambient +2.2°C), most species/populations combinations were not negatively impacted by the ocean warming scenario (except for *Z. nigricaulis* in Perth and *A. antarctica* in Coral Bay, both of which are growing at their thermal limits). This assessment shows MHWs to be of greatest concern. For temperate species (P. *sinuosa*, P. *australis* and *A. antarctica*), there was a general trend indicating populations inhabiting warmer regions are at greater risk to MHWs than populations growing in cooler regions (Table 3). This was also the case for *Z. nigricaulis*, albeit this species was only tested at one location reflective of its warmest growing region. *H. ovalis*, a globally distributed species, was of low concern for all temperature scenarios across all climatic zones assessed. Geographe Bay was the coolest location assessed for *P. sinuosa* and can be considered as a potential refugia. For all species (*P. sinuosa*, *P. australis*, *A. antarctica* and *H. ovalis*) assessed across multiple locations, at least one population was identified with a higher T_opt_ than others, suggesting potential for resilience building (Table 3).

**Table 3:** Concern rating (low, moderate or high concern), and cause of concern (summer ambient, ocean warming or marine heatwave) for seagrass species and location sampled.

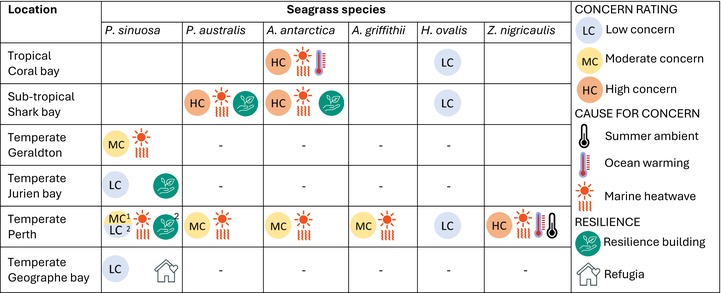

Concern ratings are based on a summer ambient, ocean warming (summer ambient + RCP6.0 2.2°C) or MHW event (based on 2010/2011), being ≥ 2°C below *T*_opt_ = low concern, 0–1.99°C below *T*_opt_ = moderate concern and higher than *T*_opt_ = high concern. A 2°C buffer has been used for concern ratings based on *T*_opt_ extracted for summer daily metabolism. Where populations have higher *T*_opt_ values than other populations, these have been recognised as options for resilience building. Refugia are those with high *T*_opt_ values in cooler regions. Blank squares indicate a species does not grow in a location, and dashed lines indicate the species grows in a location but was not sampled.

1. Seagrass populations in Perth within Shoalwater are of moderate concern.

2. Seagrass populations in Perth within Cockburn Sound (Southern Flats, Garden Island and Woodman) are of low concern and these meadows can be considered as thermal resilience building options.

### Productivity

The maximum net productivity (NP_max_) at *T*_opt_ varied among species in the temperate location (Perth), sub‐tropical location (Shark Bay) and the tropical location (Coral Bay). The overall trend showed that the smaller species (*Z. nigricaulis* and *H. ovalis*) had a 1.5‐ to 2‐fold higher NP_max_ than the larger species (*P. sinuosa*, *P. australis* and *A. antarctica*; Table 2). When comparing seagrass species (*P. australis*, *A. antarctica*, *P. sinuosa and H. ovalis*) across locations over a latitudinal gradient, NP_max_ at T_opt_ generally had a twofold increase at the warmest location assessed, except for *P. australis* which had a lower NP_max_ at the warmer location (Table 2). NP_max_ at the local scale also showed variation, with the three sites in Cockburn Sound having a *c*. 1.5 lower NP_max_ at *T*_opt_ compared with Shoalwater in Warnbro Sound.

## Discussion

Understanding variation in thermal tolerance across different species and for multiple populations of the same species is critical for accurately predicting climate change impacts and implementing restoration or resilience‐building actions (Araujo & Peterson, 2012; Bennett *et al*., 2019). This study assessed the thermal tolerance of six seagrass species, across a range of life‐history strategies (colonising, opportunistic and persistent). Additionally, the thermal tolerance of multiple populations for four species was assessed across a broad latitudinal gradient (*c*. 500–1000 km) with one species at a local scale (*c*. 25 km). The three major findings were that (1) thermal optima varied by 10°C between the six species; (2) thermal optima among populations of each single species differed by up to 4°C across a latitudinal gradient, but there was not a consistent pattern with latitude; and (3) thermal optima varied by up to 4°C at the local‐spatial scale. This demonstrates that not all species or populations within species face the same level of risk from climate change and that a one‐size‐fits‐all approach is not appropriate for management of thermally vulnerable seagrass species.

### Thermal tolerance

#### Thermal optima varies among species

A major gap in our knowledge to inform future climate predictions for plants is our limited understanding of thermal tolerances across multiple species (Donelson *et al*., 2019; Geange *et al*., 2021). This study has highlighted clear differences between seagrass species' *T*_opt_ values, which broadly coincided with their geographic thermal distributions. *Halophila ovalis* has a global distribution and was the species with the highest *T*_opt_ in this study. *Zostera nigricaulis*, the species with the most geographically restricted distribution in this study (temperate waters only), had the lowest *T*_opt_. This trend aligns with broader plant studies (Zhu *et al*., 2018), as well as seagrasses in the Mediterranean, where Bennett *et al*. (2022 b) found differences of up to 10°C in *T*_opt_ when comparing species with a broad thermal range (*Cymodocea nodosa*), to species restricted to temperate areas (*Posidonia oceanica*). Similarly, within Australia, tropical species like *Cymodocea serrulata* and *Halodule uninervis* had a higher *T*_opt_ (35°C) than *Zostera muelleri* (31°C), a predominantly temperate species (Collier *et al*., 2017). This suggests that species have a *T*_opt_ adapted to the water temperature across their distributional range, and potentially have retained their ancestral thermal traits, which has been demonstrated in terrestrial plants (O'Sullivan *et al*., 2017; Lancaster & Humphreys, 2020). Several evolutionary theories may explain how differences in *T*_opt_ and thermal ranges arise, and given the divergent evolutionary lineages of seagrasses, a variety of relationships – both among and within species – may exist between thermal range, *T*_opt_ and performance (Wooliver *et al*., 2022).

#### Thermal optima varies within a species (across locations), with no pattern across a latitudinal gradient or climatic zones

Broad scale thermal assessments of how plants will respond to climate impacts generally rely on a species' realised thermal niche, or extrapolate localised performance data across a species range, typically overlooking variation in local‐scale thermal physiology (Araujo & Peterson, 2012). Notably, findings from our study demonstrate that *T*_opt_ varies across locations for a single species, and the change in *T*_opt_ does not consistently follow latitudinal or thermal gradients, even at warm‐range edges. Consistent with our results, Sentinella *et al*. (2020) found no latitudinal trend in the thermal tolerance for seed germination across 62 plant species, suggesting that thermal adaptation may not align predictably with geographic distribution.

In our study, three seagrass species, *P. sinuosa*, *P. australis* and *A. antarctica*, were sampled from their warm‐edge and cooler southern waters (but not at their cool‐edge). Among them, *P. australis* was the only species in this study to exhibit a higher *T*_opt_ at its warm edge. However, this finding should be interpreted cautiously, as only two populations were sampled, and the Shark Bay population has recently been reclassified as a hybrid involving an unconfirmed *Posidonia* species (Edgeloe *et al*., 2022). Both *A. antarctica* and *P. sinuosa* had a lower *T*_opt_ at the warm edge of their range (Coral Bay and Geraldton, respectively). Notably, ‘*P. australis’* occupies both shallow and deep habitats at its warm edge in Shark Bay, whereas the warm‐edge populations of *A. antarctica* and *P. sinuosa* are limited to deeper (*c*. 8 m) waters (note that *A. antarctica* is only found within a small area of Coral Bay). Further south (in cooler water) where *A. antarctica* and *P. sinuosa* are more prevalent, they are dominant in both shallow and deeper waters (Shark Bay and Geraldton, respectively). Since deeper waters are cooler and more thermally stable than shallow waters, it is possible that these warm‐edge populations of *P. sinuosa* and *A. antarctica* can only persist in cooler deeper waters (Giraldo‐Ospina *et al*., 2020), possibly explaining the lower *T*_opt_ seen at the warm edge for these species. Only, *P. sinuosa* was collected at the coolest location in this study, Geographe Bay. Although this is not the species' cool edge, it experiences temperatures similar to those of the Southern‐Australian region (*P. sinuosa* cool‐edge). At this coolest location tested, *P. sinuosa* exhibited a *T*_opt_ higher than that of the warm‐edge population and more similar to the warmer location (Jurien Bay), where *P. sinuosa* is more abundant. Previous hypotheses suggest that warm‐edge populations would have the highest thermal tolerance where a species has reached its maximum adaptation and/or plasticity potential (Donelson *et al*., 2019). This study contradicts this assumption and further, is consistent with Bennett *et al*. (2022a), where *P*. *oceanica* at its cool edge had a higher tolerance to warming than the core population and was more similar to that of the warm‐edge population (Wiens *et al*., 2010; Bennett *et al*., 2022a). While the underlying cause of this pattern remains unclear, one explanation could be the retention of ancestral thermal traits shaped by historical climatic conditions, resulting in edge populations with thermal tolerances that do not align with their current local environments (Hewitt, 2004; Hampe & Petit, 2005). This key finding suggests that thermal performance does not necessarily reflect the thermal geography of a species range, an important consideration for predicting impacts from future climate scenarios.

#### Thermal optima varies at the local scale

Temperature tolerances of most species across microhabitats at local scales (10's km) are relatively understudied, especially when compared to broader‐scale assessments (100's km; Geange *et al*., 2021). Our results demonstrate that even at the local scale (within *c*. 25 km), where ambient temperature is similar, *T*_opt_ of an individual species can vary. For instance, the *T*_opt_ of *P. sinuosa* across sites within Cockburn Sound (Sites 1, 2 and 3) were similar (*T*_opt_ 30–31°C), but the *T*_opt_ of the nearby Shoalwater population was *c*. 4°C lower (26.5°C). The local oceanographic conditions and habitat type might help explain this variation, as the latter site is more regularly exposed to southerly swells and onshore winds (Webster *et al*., 2024a), which can enhance water mixing and offshore exchange, leading to locally cooler and more stable temperatures that may contribute to lower a *T*_opt_. Alternatively, micro‐climates at the local scale (which have not been assessed), could contribute to the observed differences in *T*_opt_. Micro‐climates or local‐scale (2–12 km) environmental pressures (e.g. light and grazing) in an estuarine environment for *Zostera marina* have been shown to increase phenotypic variation among neighbouring populations, and notably, this transcended into some populations of *Z. marina* exhibiting a higher tolerance to warmer temperatures at the local scale (DuBois *et al*., 2022). Similar patterns showing preadapted populations at the local scale have been observed in corals (Bay & Palumbi, 2014). Local‐scale differences in *T*_opt_ could be positive for a species to persist within an area under changing environmental conditions (DuBois *et al*., 2022). However, it is also important to consider that selection for populations with higher *T*_opt_ could have trade‐offs in relation to ecosystem function (Reynolds *et al*., 2012). In this study, we found that not all seagrass habitat had equal value. For instance, net productivity was 1.5‐fold higher in the Shoalwater population, which had the lowest *T*_opt_ (most at risk from ocean warming and MHW impacts), compared to neighbouring populations with higher *T*_opt_ values. Furthermore, if heat tolerant genotypes are selected, this may lead to reduced population genetic diversity within meadows and potentially reduced adaptive capacity to other pressures (Vásquez *et al*., 2023). Additional research is warranted at the local‐spatial scale for other seagrass species, as well as at the individual scale (individual plants within a meadow), ideally with corresponding genetic and environmental data to further decipher potential mechanisms (e.g. environment or genotype) influencing *T*_opt_.

### Thermal vulnerability assessment across and within species for current and future climate scenarios

To implement effective conservation strategies in the face of climate change, it is crucial to identify which species and populations are most vulnerable to temperature‐related impacts. Based on vulnerability metrics (‘Thermal vulnerability assessment’ in the Materials and Methods section) applied in this study, we found that most species and populations are currently experiencing ambient summer temperatures below their *T*_opt_. The only exception to this was *Z. nigricaulis* which predominantly occurs in southern Australia in cooler waters (ALA, 2025). Under a future warming scenario (summer +2.2°C), our assessment suggests that most species and populations (except for *Z. nigricaulis* in Perth and *A. antarctica* in Coral Bay) will benefit from warming, with an increase in productivity at higher temperatures. Even though ocean warming may lead to increased productivity for some species, it is important to consider that increased water temperature may make seagrasses more vulnerable to other stressors, such as sulphide intrusion and light reduction (Pedersen *et al*., 2004; Collier & Waycott, 2014; Brodersen *et al*., 2017). Based on this, we hypothesise that under a warming scenario of 2.2°C, *A. antarctica* and *Z. nigricaulis* may experience a range contraction at their warm edge. By contrast, since the other species assessed in this study are already growing in cooler waters, their ranges are unlikely to be significantly affected (Chen *et al*., 2011). MHWs were the predominant threat to temperate seagrass species in this study, particularly those in tropical climatic zones where they inhabit areas closer to their upper thermal limits (Sentinella *et al*., 2020). It is important to note that the MHW scenario we included in this study was based on the temperatures observed in the well‐documented 2010/2011 MHW event in WA (Pearce & Feng, 2013). If future MHWs of similar or greater intensity (than that of the 2010/2011 MHW) occur in temperate regions as predicted (Oliver *et al*., 2018), then the risk to temperate regions will likely be elevated (Jung *et al*., 2023; Booth *et al*., 2024). This approach of using *T*_opt_ data to identify thermally vulnerable areas is critical for targeted local management. While global efforts to reduce greenhouse gas emissions are essential to mitigate climate change, local management actions in thermally vulnerable areas – such as reducing coastal development or nutrient runoff – are critical for alleviating cumulative stressors that compound climate impacts (McMahon *et al*., 2022).

In addition to intensity, the duration of a MHW is also a critical determinant of its ecological impact (Oliver *et al*., 2019). Seagrass resistance and recovery can broadly be classified according to their life‐history strategy – colonising, opportunistic or persistent (Kilminster *et al*., 2015) – with visible decline in colonising genera, such as *Halophila* occurring within days to weeks, whereas persistent genera like *Posidonia*, may take months to exhibit visible decline (Collier & Waycott, 2014; Webster *et al*., 2024 b). During the 2010/2011 MHW in Shark Bay, temperatures reached 2–5°C above summer averages (28–31°C), resulting in the loss of *c*. 24% (1310 km^2^) of the region's dense seagrass area. Notably, *A. antarctica* was more severely impacted than *P. australis*, despite both being dominant species in the area (Strydom *et al*., 2020). While the *T*_opt_ for *A. antarctica* (29.5°C) was *c*. 1°C lower than that of *P. australis* (30.5°C) at Middle Bluff, Shark Bay, it is important to acknowledge that this small difference in *T*_opt_ alone does not fully explain the contrasting resilience observed between these species. The greater losses of *A. antarctica* following the 2010/2011 MHW likely reflect a combination of physiological, morphological and life‐history traits rather than sole differences in TPCs (Kilminster *et al*., 2015). Specifically, the persistent nature of *Posidonia*, with its larger carbohydrate reserves and extensive belowground biomass, provides greater buffering capacity against disturbances compared with the opportunistic/persistent growth form of *Amphibolis* (Kilminster *et al*., 2015; Strydom *et al*., 2020; Said *et al*., 2024).

Our vulnerability assessment has been based on physiological *T*_opt_ data, and while temperatures close to *T*_opt_ may afford the plants some buffering capacity at the physiological scale, under a prolonged MHW event, it is possible that at the plant scale carbohydrates stores will be reduced and the overall resilience to further stressors compromised. Therefore, it is likely that if short‐term experiments in this study were run over a longer duration the *T*_opt_ and CT_max_ would be lower. Notably, the *T*_opt_ for photosynthesis is often higher than that for growth (Lee *et al*., 2007), which may explain why physiological decline or mortality often occurs at or just above the *T*_opt_ for photosynthesis (Collier *et al*., 2018; Campbell & Le, 2025). For example, *Zostera muelleri*, with a physiological *T*_opt_ of 31°C (Collier *et al*., 2017), exhibited near complete mortality after 1 month of exposure to 33°C under mesocosm conditions (Collier *et al*., 2011). For assessments of growth or survival, a more ecologically meaningful threshold may be the temperature at which 50% of individuals experience mortality (LT50; Jiang *et al*., 2022; Savva *et al*., 2018). Within this context, we consider the vulnerability assessment undertaken in this study to be ecologically meaningful for three primary reasons. First, to assess vulnerability to climate impacts we have used *T*_opt_ as thermal cut‐off, rather than CT_max_. We do not advocate for the physiological CT_max_ to be used in a management context (Collier *et al*., 2016), as this acute thermal threshold typically leads to *c*. 50% mortality within minutes to hours and does not reflect the stress response relevant to longer term (days to weeks) exposure (Rinaldi *et al*., 2023). Second, we have used whole plants, which take into consideration both photosynthetic and respiratory demand, which can alter *T*_opt_ (Collier *et al*., 2017). Third, we acknowledge that the rapid increase in temperature in this study does not necessarily reflect the gradual onset of a MHW event, or the potential for plants to acclimate if they were exposed to higher temperatures over a longer period of time (Lambers & Oliveira, 2019). However, previous research using a similar approach to calculate *T*_opt_ (Collier *et al*., 2011) has demonstrated that sustained exposure to temperatures beyond *T*_opt_ values for extended periods of up to 4 wk is ecologically relevant for assessing seagrass vulnerability. To further account for this uncertainty, we incorporated assessments of daily plant metabolism alongside instantaneous NP rates, and applied a conservative 2°C buffer into our risk ratings (as per concern ratings in ‘Thermal vulnerability assessment’ in the Materials and Methods section, and built into Tables 2, 3).

### Resilience‐building opportunities

To date, strategies for protecting seagrass have focused on either protecting existing meadows or restoring degraded meadows to their historical state (Unsworth *et al*., 2024). While these strategies are valuable, conservation efforts need to extend into actions where resilience to projected climate change scenarios is incorporated (e.g. Coleman *et al*., 2020; Gaitán‐Espitia & Hobday, 2020). There is a range of actions that can be taken to build resilience to future pressures, such as genetic rescue, assisted gene flow and assisted evolution using breeding or biotechnology (e.g. Caruso *et al*., 2021; Coleman & Bragg, 2021), but to our knowledge, none of these have been trialled for seagrasses. This study revealed that for multiple species (*P. australis*, *P. sinuosa*, *A. antarctica* and *H. ovalis*), there are populations that have a higher *T*_opt_ which could make them ‘better equipped’ to deal with thermal pressures. Even though there was no clear pattern for higher *T*_opt_ at the warmest edge of the species range (*P. australis*, *P. sinuosa* and *A. antarctica*), for all species there was a seagrass population within a warmer location that had a higher *T*_opt_ (Tables 2, 3). Assuming the differences in *T*_opt_ are adaptive, the resilience‐building strategy of assisted gene flow could be employed where these ‘preadapted individuals’ are introduced to areas within the existing distributional range to facilitate adaptation to future conditions. These populations could be targeted as source populations for restoration or assisted migration to areas poleward, to future‐proof these meadows against warming (Pickup *et al*., 2012; Aitken & Whitlock, 2013). This approach could be incorporated into existing restoration programmes, such as Seeds for Snapper or Operation Posidonia. Opportunities for resilience building based on the results of this study are identified in Table 3. However, further trials are required to confirm whether populations will retain a higher *T*_opt_ when translocated into cooler waters. Another important consideration before implementing these actions is the level of genetic connectivity between source and translocated populations (Gaitán‐Espitia & Hobday, 2020). If the populations are genetically distinct, there is the risk of outbreeding depression or the disruption of locally adapted genotypes (Twardek *et al*., 2023).

### Conclusion

Our study provides a thermal vulnerability assessment of future warming and MHWs for six seagrass species, evaluating multiple populations across both broad (100's km) and local (10's km) scales to guide conservation and resilience‐building efforts. We conclude that most species assessed in this study may benefit from small increases in temperature; however, MHWs are likely to have negative implications. In particular, populations of temperate species occurring in sub‐tropical and tropical climates face greater risks from MHWs, as they are already growing near their upper thermal limits. Only one globally distributed species, *H. ovalis*, was assessed in this study, and it was the only species in this study that was predicted to be unimpacted from climate impacts across temperate to tropical regions. However, intensifying MHWs in tropical regions may pose future challenges for *H. ovalis*. Additionally, there is evidence to suggest that *T*_opt_ varies at local scales. While local‐scale variability can enhance the persistence of heat‐tolerant populations of a species in an area, it may also have negative implications for genetic diversity and ecosystem function. It is further important to note that thermal performance does not necessarily reflect the thermal geography of a seagrass species range. Therefore, it is essential to quantify *T*_opt_ both within and across species and at both local and broad scales to more accurately predict and manage future climate impacts.

## Competing interests

None declared.

## Author contributions

NS was responsible for conceptualisation, data curation, formal analysis, funding acquisition, investigation, methodology and project administration; and contributed to the writing of the original draft and review and editing. CW acquired funding and performed investigation, and assisted with writing – review and editing. SS contributed to conceptualization and funding acquisition, and assisted with writing – review and editing. ND contributed to the investigation. MPA provided software and assisted with writing – review and editing. KM contributed to conceptualization and funding acquisition, participated in supervision and assisted with writing – review and editing.

## Disclaimer

The New Phytologist Foundation remains neutral with regard to jurisdictional claims in maps and in any institutional affiliations.

## Supporting information


**Fig. S1** Experimental setup for testing seagrass thermal tolerance under light and dark conditions.
**Fig. S2** Metabolic rate change (net photosynthesis Q_10_) below optimum temperature across seagrass species and sites.
**Table S1** Collection location, date, and coordinates for each seagrass species.
**Table S2** Chamber volume and orientation for experimental conditions.
**Table S3** Gross photosynthesis and respiration for seagrass species and locations.
**Table S4** Whole plant metabolism net photosynthesis derived per day for species and locations.
**Table S5** Differences in thermal optima between daily plant metabolism and hourly net photosynthesis.
Please note: Wiley is not responsible for the content or functionality of any Supporting Information supplied by the authors. Any queries (other than missing material) should be directed to the *New Phytologist* Central Office.

## Data Availability

All data are available within the article and Tables 2, S3 and S4.

## References

[nph70742-bib-0001] Adams MP , Collier CJ , Uthicke S , Ow YX , Langlois L , O'Brien KR . 2017. Model fit versus biological relevance: evaluating photosynthesis‐temperature models for three tropical seagrass species. Scientific Reports 7: 39930. 28051123 10.1038/srep39930 PMC5209739

[nph70742-bib-0002] Aitken SN , Whitlock MC . 2013. Assisted gene flow to facilitate local adaptation to climate change. Annual Review of Ecology, Evolution, and Systematics 44: 367–388.

[nph70742-bib-0003] Araujo MB , Peterson AT . 2012. Uses and misuses of bioclimatic envelope modelling. Ecology 93: 1527–1539. 22919900 10.1890/11-1930.1

[nph70742-bib-0004] Arias‐Ortiz A , Serrano O , Masqué P , Lavery PS , Mueller U , Kendrick GA , Rozaimi M , Esteban A , Fourqurean JW , Marbà N *et al*. 2018. A marine heatwave drives massive losses from the world's largest seagrass carbon stocks. Nature Climate Change 8: 338–344.

[nph70742-bib-0005] Atkin OK , Tjoelker MG . 2003. Thermal acclimation and the dynamic response of plant respiration to temperature. Trends in Plant Science 8: 343–351. 12878019 10.1016/S1360-1385(03)00136-5

[nph70742-bib-0006] Atlas of Living Australia (ALA) . 2025. Heterozostera nigricaulis J.Kuo . [WWW document] URL https://bie.ala.org.au/species/https://id.biodiversity.org.au/node/apni/2890411.

[nph70742-bib-0007] Baird ME , Adams MP , Babcock RC , Oubelkheir K , Mongin M , Wild‐Allen KA , Skerratt J , Robson BJ , Petrou K , Ralph PJ *et al*. 2016. A biophysical representation of seagrass growth for application in a complex shallow‐water biogeochemical model. Ecological Modelling 325: 13–27.

[nph70742-bib-0008] Baldock J , Bancroft KP , Williams M , Shedrawi G , Field S . 2014. Accurately estimating local water temperature from remotely sensed satellite sea surface temperature: a near real‐time monitoring tool for marine protected areas. Ocean and Coastal Management 96: 73–81.

[nph70742-bib-0009] Ban NC , Whitney C , Davies TE , Buscher E , Lancaster D , Eckert L , Rhodes C , Jacob AL . 2017. Chapter 8 – Conservation actions at global and local scales in marine social – ecological systems: status, gaps, and ways forward. In: Conservation for the Anthropocene Ocean. London: Elsevier, 143–168. doi: 10.1016/B978-0-12-805375-1.00008-8.

[nph70742-bib-0010] Barley JM , Cheng BS , Sasaki M , Gignoux‐Wolfsohn S , Hays CG , Putnam AB , Sheth S , Villeneuve AR , Kelly M . 2021. Limited plasticity in thermally tolerant ectotherm populations: evidence for a trade‐off. Proceedings of the Royal Society B: Biological Sciences 288: 20210765. 10.1098/rspb.2021.0765 PMC8424342 34493077

[nph70742-bib-0011] Bates OK , Bertelsmeier C . 2021. Climatic niche shifts in introduced species. Current Biology 31: R1252–R1266. 34637738 10.1016/j.cub.2021.08.035

[nph70742-bib-0012] Bay RA , Palumbi SR . 2014. Report multilocus adaptation associated with heat resistance in reef‐building corals. Current Biology 24: 2952–2956. 25454780 10.1016/j.cub.2014.10.044

[nph70742-bib-0013] Bennett JM , Calosi P , Clusella‐Trullas S , Martínez B , Sunday J , Algar AC , Araújo MB , Hawkins BA , Keith S , Kühn I *et al*. 2018. Data Descriptor: GlobTherm, a global database on thermal tolerances for aquatic and terrestrial organisms. Scientific Data 5: 180022. 29533392 10.1038/sdata.2018.22 PMC5848787

[nph70742-bib-0014] Bennett S , Alcoverro T , Kletou D , Antoniou C , Boada J , Cucala L , Jorda G , Kleitou P , Roca G , Santana‐garcon J *et al*. 2022a. Resilience of seagrass populations to thermal stress does not reflect regional differences in ocean climate. New Phytologist 233: 1657–1666. 34843111 10.1111/nph.17885 PMC9299911

[nph70742-bib-0015] Bennett S , Duarte CM , Marba N , Wernberg T . 2019. Integrating within‐species variation in thermal physiology into climate change ecology. Philosophical Transactions of the Royal Society of London. Series B: Biological Sciences 374: 20180550. 31203756 10.1098/rstb.2018.0550 PMC6606463

[nph70742-bib-0016] Bennett S , Vaquer‐sunyer R , Jordá G , Forteza M , Roca G , Marbà N , Donelson JM . 2022b. Thermal performance of seaweeds and seagrasses across a regional climate gradient. Frontiers in Marine Science 9: 1–11.35450130

[nph70742-bib-0017] Booth MW , Sinclair EA , Jung EMU , Austin R , Bayer PE , Krauss SL , Breed MF , Kendrick GA . 2024. Comparative gene co‐expression networks show enrichment of brassinosteroid and vitamin B processes in a seagrass under simulated ocean warming and extreme climatic events. Frontiers in Plant Science 15: 1309956. 38344183 10.3389/fpls.2024.1309956 PMC10853371

[nph70742-bib-0018] Brodersen KE , Hammer KJ , Schrameyer V , Floytrup A , Rasheed MA , Ralph PJ , Kühl M , Pedersen O . 2017. Sediment resuspension and deposition on seagrass leaves impedes internal plant aeration and promotes phytotoxic H2S intrusion. Frontiers in Plant Science 8: 657. 28536583 10.3389/fpls.2017.00657 PMC5423392

[nph70742-bib-0019] Campbell ML , Le CTU . 2025. Varying vulnerabilities: seagrass species under threat from prolonged ocean warming. Limnology and Oceanography: 1–16. doi: 10.1002/lno.70156.

[nph70742-bib-0020] Caruso C , Hughes K , Drury C . 2021. Selecting heat‐tolerant corals for proactive reef restoration. Frontiers in Marine Science 8: 632027.

[nph70742-bib-0021] Chen AI , Hill JK , Ohlemüller R , Roy DB , Chris D , Hui JK , Ohlem R , Roy D , Thomas CD . 2011. Rapid range shifts of species associated with high levels of climate warming. Science 333: 1024–1026. 21852500 10.1126/science.1206432

[nph70742-bib-0022] Coleman MA , Bragg JG . 2021. A decision framework for evidence‐based climate adaptation interventions. Global Change Biology 27: 472–474. 33128838 10.1111/gcb.15429

[nph70742-bib-0023] Coleman MA , Wood G , Filbee‐Dexter K , Minne AJP , Goold HD , Vergés A , Marzinelli EM , Steinberg PD , Wernberg T . 2020. Restore or redefine: future trajectories for restoration. Frontiers in Marine Science 7: 237.

[nph70742-bib-0024] Collier C , Adams MP , Langlois L , Waycott M , O'Brien KR , Maxwell PS , McKenzie L . 2016. Thresholds for morphological response to light reduction for four tropical seagrass species. Ecological Indicators 67: 358–366.

[nph70742-bib-0025] Collier CJ , Langlois L , Ow Y , Johansson C , Giammusso M , Adams MP , O'Brien KR , Uthicke S . 2018. Losing a winner: thermal stress and local pressures outweigh the positive effects of ocean acidification for tropical seagrasses. New Phytologist 219: 1005–1017. 29855044 10.1111/nph.15234

[nph70742-bib-0026] Collier CJ , Ow YX , Langlois L , Uthicke S , Johansson CL , O'Brien KR , Hrebien V , Adams MP . 2017. Optimum temperatures for net primary productivity of three tropical seagrass species. Frontiers in Plant Science 8: 1–14. 28878790 10.3389/fpls.2017.01446 PMC5572403

[nph70742-bib-0027] Collier CJ , Uthicke S , Waycott M . 2011. Thermal tolerance of two seagrass species at contrasting light levels: implications for future distribution in the Great Barrier Reef. Limnology and Oceanography 56: 2200–2210.

[nph70742-bib-0028] Collier CJ , Waycott M . 2014. Temperature extremes reduce seagrass growth and induce mortality. Marine Pollution Bulletin 83: 483–490. 24793782 10.1016/j.marpolbul.2014.03.050

[nph70742-bib-0029] Corlett RT , Westcott DA . 2013. Will plant movements keep up with climate change? Trends in Ecology & Evolution 28: 482–488. 23721732 10.1016/j.tree.2013.04.003

[nph70742-bib-0030] Curtis EM , Gollan J , Murray BR , Leigh A . 2016. Native microhabitats better predict tolerance to warming than latitudinal macro‐climatic variables in arid‐zone plants. Journal of Biogeography 43: 1156–1165.

[nph70742-bib-0031] Diaz‐Almela E , Marbà N , Duarte CM . 2007. Consequences of Mediterranean warming events in seagrass (*Posidonia oceanica*) flowering records. Global Change Biology 13: 224–235.

[nph70742-bib-0032] Dichiera AM , Earhart ML , Bugg WS , Brauner CJ , Schulte PM . 2024. Too hot to handle: a meta‐analytical review of the thermal tolerance and adaptive capacity of North American Sturgeon. Global Change Biology 30: e17564. 39563555 10.1111/gcb.17564

[nph70742-bib-0033] Donelson JM , Sunday JM , Figueira WF , Gaitán‐Espitia JD , Hobday AJ , Johnson CR , Leis JM , Ling SD , Marshall D , Pandolfi JM *et al*. 2019. Understanding interactions between plasticity, adaptation and range shifts in response to marine environmental change. Philosophical Transactions of the Royal Society of London. Series B: Biological Sciences 374: 20180186. 30966966 10.1098/rstb.2018.0186 PMC6365866

[nph70742-bib-0034] DuBois K , Pollard KN , Kauffman BJ , Williams SL , Stachowicz JJ . 2022. Local adaptation in a marine foundation species: implications for resilience to future global change. Global Change Biology 28: 2596–2610. 35007376 10.1111/gcb.16080

[nph70742-bib-0035] Dunic JC , Brown CJ , Connolly RM , Turschwell MP , Côté IM . 2021. Long term declines and recovery of meadow area across the world's seagrass bioregions. Global Change Biology 27: 4096–4109. 33993580 10.1111/gcb.15684

[nph70742-bib-0036] Edgeloe JM , Severn‐Ellis AA , Bayer PE , Mehravi S , Breed MF , Krauss SL , Batley J , Kendrick GA , Sinclair EA . 2022. Extensive polyploid clonality was a successful strategy for seagrass to expand into a newly submerged environment. Proceedings of the Royal Society B: Biological Sciences 289: 20220538. 10.1098/rspb.2022.0538 PMC9156900 35642363

[nph70742-bib-0037] Feeley K , Martinez‐villa J , Perez T , Duque AS . 2020. The thermal tolerances, distributions, and performances of tropical montane tree species. Frontiers in Forests and Global Change 3: 1–11.

[nph70742-bib-0038] Gaitán‐Espitia JD , Hobday AJ . 2020. Evolutionary principles and genetic considerations for guiding conservation interventions under climate change. Global Change Biology 27: 475–488. 32979891 10.1111/gcb.15359

[nph70742-bib-0039] Geange SR , Arnold PA , Catling AA , Coast O , Cook AM , Gowland KM , Leigh A , Notarnicola RF , Posch BC , Venn SE *et al*. 2021. The thermal tolerance of photosynthetic tissues: a global systematic review and agenda for future research. New Phytologist 229: 2497–2513. 33124040 10.1111/nph.17052

[nph70742-bib-0040] Giraldo‐Ospina A , Kendrick GA , Hovey RK . 2020. Depth moderates loss of marine foundation species after an extreme marine heatwave: could deep temperate reefs act as a refuge? Proceedings of the Royal Society B: Biological Sciences 287: 20200709. 10.1098/rspb.2020.0709 PMC7341917 32517616

[nph70742-bib-0041] Halpern BS , Frazier M , Afflerbach J , Lowndes JS , Micheli F , Hara CO , Scarborough C , Selkoe KA . 2019. Recent pace of change in human impact on the world's ocean. Scientific Reports 9: 11609. 31406130 10.1038/s41598-019-47201-9 PMC6691109

[nph70742-bib-0042] Hampe A , Petit R . 2005. Conserving biodiversity under climate change: the rear edge matters. Ecology Letters 8: 461–467. 21352449 10.1111/j.1461-0248.2005.00739.x

[nph70742-bib-0043] Hewitt GM . 2004. Genetic consequences of climatic oscillations in the Quaternary. The Royal Society 359: 183–195. 10.1098/rstb.2003.1388 PMC1693318 15101575

[nph70742-bib-0044] Hobday AJ , Pecl GT . 2014. Identification of global marine hotspots: sentinels for change and vanguards for adaptation action. Reviews in Fish Biology and Fisheries 24: 415–425.

[nph70742-bib-0045] IPCC . 2023. Summary for policymakers. In: Core Writing Team , Lee H , Romero J , eds. Climate change 2023: synthesis report. Contribution of working groups I, II and III to the sixth assessment report of the Intergovernmental Panel on Climate Change. Geneva, Switzerland: IPCC, 1–34. doi: 10.59327/IPCC/AR6-9789291691647.001.

[nph70742-bib-0046] Jiang WY , Zhang YH , Liu YC , Li WT , Xu JG , Zhang PD . 2022. The effect of abrupt increase in water temperature on the survival and growth of eelgrass *Zostera marina* . Aquatic Botany 183: 103572.

[nph70742-bib-0048] Jung EMU , Abdul Majeed NAB , Booth MW , Austin R , Sinclair EA , Fraser MW , Martin BC , Oppermann LMF , Bollen M , Kendrick GA . 2023. Marine heatwave and reduced light scenarios cause species‐specific metabolomic changes in seagrasses under ocean warming. New Phytologist 239: 1692–1706. 37357353 10.1111/nph.19092

[nph70742-bib-0049] Kajtar JB , Holbrook NJ , Hernaman V . 2021. A catalogue of marine heatwave metrics and trends for the Australian region. Journal of Southern Hemisphere Earth Systems Science 71: 284–302.

[nph70742-bib-0050] Kilminster K , McMahon K , Waycott M , Kendrick G , Scanes P , McKenzie L , O'Brien KR , Lyons M , Ferguson A , Maxwell P *et al*. 2015. Unravelling complexity in seagrass systems for management: Australia as a microcosm. Science of the Total Environment 534: 97–109. 25917445 10.1016/j.scitotenv.2015.04.061

[nph70742-bib-0051] Lambers H , Oliveira RS . 2019. Plant physiological ecology, 3^rd^ edn. Cham, Switzerland: Springer Nature Switzerland AG. 10.1007/978-3-030-29639-1.

[nph70742-bib-0052] Lancaster LT , Humphreys AM . 2020. Global variation in the thermal tolerances of plants. Proceedings of the National Academy of Sciences, USA 117: 13580–13587. 10.1073/pnas.1918162117 PMC7306813 32482870

[nph70742-bib-0053] Lee KS , Park SR , Kim YK . 2007. Effects of irradiance, temperature, and nutrients on growth dynamics of seagrasses: a review. Journal of Experimental Marine Biology and Ecology 350: 144–175.

[nph70742-bib-0054] Marbà N , Duarte CM . 2010. Mediterranean warming triggers seagrass (*Posidonia oceanica*) shoot mortality. Global Change Biology 16: 2366–2375.

[nph70742-bib-0055] Marba N , Jordà G , Bennett S , Duarte CM . 2022. Seagrass thermal limits and vulnerability to future warming. Frontiers in Marine Science 9: 1–10.35450130

[nph70742-bib-0056] McMahon K , Kilminster K , Canto R , Roelfsema C , Lyons M , Kendrick GA , Waycott M , Udy J . 2022. The risk of multiple anthropogenic and climate change threats must be considered for continental scale conservation and management of seagrass habitat. Frontiers in Marine Science 9: 1–15.35450130

[nph70742-bib-0100] Nievola CC , Carvalho CP , Carvalho V , Rodrigues E . 2017. Rapid responses of plants to temperature changes. Temperature 4: 371–405. 10.1080/23328940.2017.1377812 PMC5800372 29435478

[nph70742-bib-0057] O'Sullivan OS , Heskel MA , Reich PB . 2017. Thermal limits of leaf metabolism across biomes. Global Change Biology 23: 209–223. 27562605 10.1111/gcb.13477

[nph70742-bib-0058] O2 Marine . 2021. Port Geographe Marina Monitoring Program (2016–2021). Close‐out Report.

[nph70742-bib-0059] Oliver ECJ , Burrows MT , Donat MG , Sen Gupta A , Alexander LV , Perkins‐Kirkpatrick SE , Benthuysen JA , Hobday AJ , Holbrook NJ , Moore PJ *et al*. 2019. Projected marine heatwaves in the 21st century and the potential for ecological impact. Frontiers in Marine Science 6: 734.

[nph70742-bib-0060] Oliver ECJ , Donat MG , Burrows MT , Moore PJ , Smale DA , Alexander LV , Benthuysen JA , Feng M , Gupta AS , Hobday AJ *et al*. 2018. Longer and more frequent marine heatwaves over the past century. Nature Communications 9: 1324. 10.1038/s41467-018-03732-9 PMC5893591 29636482

[nph70742-bib-0061] Ontoria Y , Webster C , Said N , Ruiz JM , Pérez M , Romero J , McMahon K . 2020. Positive effects of high salinity can buffer the negative effects of experimental warming on functional traits of the seagrass *Halophila ovalis* . Marine Pollution Bulletin 158: 111404. 32753189 10.1016/j.marpolbul.2020.111404

[nph70742-bib-0062] Orth RJ , Carruthers TJB , Dennison WC , Duarte CM , Fourqurean JW , Heck KL , Randall Hughes A , Kendrick G , Kenworthy WJ , Olyarnik S *et al*. 2006. A global crisis for seagrass ecosystems. Bioscience 56: 987.

[nph70742-bib-0063] Pearce AF , Feng M . 2013. The rise and fall of the “marine heat wave” off Western Australia during the summer of 2010/2011. Journal of Marine Systems 111: 139–156.

[nph70742-bib-0064] Pedersen O , Binzer T , Borum J . 2004. Sulphide intrusion in eelgrass (*Zostera marina* L.). Plant, Cell & Environment 27: 595–602.

[nph70742-bib-0065] Pedersen O , Colmer TD , Borum J , Zavala‐Perez A , Kendrick G . 2016. Heat stress of two tropical seagrass species during low tides – impact on underwater net photosynthesis, dark respiration and diel *in situ* internal aeration. New Phytologist 210: 1207–1218. 26914396 10.1111/nph.13900

[nph70742-bib-0066] Pickup M , Field DL , Rowell DM , Young AG . 2012. Predicting local adaptation in fragmented plant populations: implications for restoration genetics. Evolutionary Applications 5: 913–924. 23346235 10.1111/j.1752-4571.2012.00284.x PMC3552408

[nph70742-bib-0067] Quigley KM . 2024. Breeding and selecting corals resilient to global warming. Annual Review of Animal Biosciences 12: 209–332. 37931139 10.1146/annurev-animal-021122-093315

[nph70742-bib-0068] Ralph PJ , Burchett MD . 1995. Photosynthetic responses of the seagrass *Halophila ovalis* (R. Br.) Hook. f. to high irradiance stress, using chlorophyll a fluorescence. Aquatic Botany 51: 55–66.

[nph70742-bib-0069] Reynolds LK , McGlathery KJ , Waycott M . 2012. Genetic diversity enhances restoration success by augmenting ecosystem services. PLoS ONE 7: 1–7. 10.1371/journal.pone.0038397 PMC3382623 22761681

[nph70742-bib-0070] Rinaldi A , Martinez M , Badalamenti F , Anna GD , Mirto S , Mar L , Procaccini G , Montalto V . 2023. The ontogeny‐specific thermal sensitivity of the seagrass *Posidonia oceanica* . Frontiers in Marine Science 10: 1183728.

[nph70742-bib-0071] Ruggeri M , Million WC , Hamilton L , Kenkel CD . 2024. Microhabitat acclimatization alters sea anemone – algal symbiosis and thermal tolerance across the intertidal zone. Ecology 105: e4388. 39076113 10.1002/ecy.4388

[nph70742-bib-0072] Said N , Webster C , Dunham N , Strydom S , McMahon K . 2024. Current state of knowledge for dredging and climate change impacts on seagrass ecosystems to inform environmental impact assessment and management. A case study: Cockburn Sound and Owen Anchorage. Prepared for the WAMSI Westport Marine Science Program. Western Australian Marine Science Institution, Perth, Western Australia.

[nph70742-bib-0073] Savva I , Bennett S , Roca G , Jordà G , Marbà N . 2018. Thermal tolerance of Mediterranean marine macrophytes: vulnerability to global warming. Ecology and Evolution 8: 12032–12043. 30598797 10.1002/ece3.4663 PMC6303755

[nph70742-bib-0074] Sentinella AT , Warton DI , Sherwin WB , Offord CA , Moles AT . 2020. Tropical plants do not have narrower temperature tolerances, but are more at risk from warming because they are close to their upper thermal limits. Global Ecology and Biogeography 29: 1387–1398.

[nph70742-bib-0075] Somero GN . 2010. The physiology of climate change: how potentials for acclimatization and genetic adaptation will determine “winners” and “losers”. Journal of Experimental Biology 213: 912–920. 20190116 10.1242/jeb.037473

[nph70742-bib-0076] Strydom S , Murray K , Wilson S , Huntley B , Rule M , Heithaus M , Bessey C , Kendrick GA , Burkholder D , Fraser MW *et al*. 2020. Too hot to handle: unprecedented seagrass death driven by marine heatwave in a World Heritage Area. Global Change Biology 26: 3525–3538. 32129909 10.1111/gcb.15065

[nph70742-bib-0077] Sumner EE , Williamson VG , Gleadow RM , Wevill T , Venn SE . 2022. Acclimation to water stress improves tolerance to heat and freezing in a common alpine grass. Oecologia 199: 831–843. 35974110 10.1007/s00442-022-05245-1 PMC9464112

[nph70742-bib-0078] Trisos CH , Merow C , Pigot AL . 2020. The projected timing of abrupt ecological disruption from climate change. Nature 580: 496–501. 32322063 10.1038/s41586-020-2189-9

[nph70742-bib-0079] Twardek WM , Taylor JJ , Rytwinski T , Aitken SN , Macdonald AL , Van Bogaert R , Cooke SJ . 2023. The application of assisted migration as a climate change adaptation tactic: an evidence map and synthesis. Biological Conservation 280: 109932.

[nph70742-bib-0080] Unsworth RKF , Jones BLH , Bertelli CM , Coals L , Cullen‐Unsworth LC , Mendzil AF , Rees SC , Taylor F , Walter B , Evans AJ . 2024. Ten golden rules for restoration to secure resilient and just seagrass social‐ecological systems. Plants, People, Planet 7: 33–48.

[nph70742-bib-0081] Vásquez C , Quiñones RA , Brante A , Hernández‐Miranda E . 2023. Genetic diversity and resilience in benthic marine populations. Revista Chilena de Historia Natural 96: 1–10.

[nph70742-bib-0082] Verberk WCEP , Hoefnagel KN , Peralta‐Maraver I , Floury M , Rezende EL . 2023. Long‐term forecast of thermal mortality with climate warming in riverine amphipods. Global Change Biology 29: 5033–5043. 37401451 10.1111/gcb.16834

[nph70742-bib-0083] Waycott M , Duarte CM , Carruthers TJB , Orth RJ , Dennison WC , Olyarnik S , Calladine A , Fourqurean JW , Heck KL , Hughes R *et al*. 2009. Accelerating loss of seagrasses across the globe threatens coastal ecosystems. Proceedings of the National Academy of Sciences, USA 106: 12377–12381. 10.1073/pnas.0905620106 PMC2707273 19587236

[nph70742-bib-0084] Webster C , McMahon K , Ross C , Afrifa‐Yamoah E , Said N , Martin B , Strydom S . 2024a. Two decades of seagrass monitoring data show drivers include ENSO, climate warming and local stressors . Prepared for the WAMSI Westport Marine Science Program. Western Australian Marine Science Institution, Perth, Western Australia.

[nph70742-bib-0085] Webster C , Said NE , Dunham N , Bywater A , Jung M , Billinghurst J , Strydom S , McMahon KM . 2024b. Posidonia sinuosa *tolerates the cumulative effects of a short term heatwave and low light event*. Prepared for the WAMSI Westport Marine Science Program. Western Australian Marine Science Institution, Perth, WA, Australia. 25 pp. [WWW document] URL https://wamsi.org.au/wp‐content/uploads/2024/11/WWMSP_2.2_Posidonia_sinuosa_tolerates_heatwave_and_low_light_FINAL‐1.pdf.

[nph70742-bib-0086] Wiens JJ . 2016. Climate‐related local extinctions are already widespread among plant and animal species. PLoS Biology 14: 1–18. 10.1371/journal.pbio.2001104 PMC5147797 27930674

[nph70742-bib-0087] Wiens JJ , Ackerly DD , Allen AP , Anacker BL , Buckley LB , Cornell HV , Damschen EI , Jonathan Davies T , Grytnes JA , Harrison SP *et al*. 2010. Niche conservatism as an emerging principle in ecology and conservation biology. Ecology Letters 13: 1310–1324. 20649638 10.1111/j.1461-0248.2010.01515.x

[nph70742-bib-0088] Wooliver R , Vtipilthorpe EE , Wiegmann AM , Sheth SN . 2022. A viewpoint on ecological and evolutionary study of plant thermal performance curves in a warming world. AoB Plants 14: 1–8. 10.1093/aobpla/plac016 PMC9126585 35615255

[nph70742-bib-0089] Yan W , Hunt LA . 1999. An equation for modelling the temperature response of plants using only the cardinal temperatures. Annals of Botany 84: 607–614.

[nph70742-bib-0090] Zhu L , Odhran S , Bloomfield KJ , Hocart CH , Lasantha K , Egerton JJG , Atkin OK . 2018. Plasticity of photosynthetic heat tolerance in plants adapted to thermally contrasting biomes. Plant, Cell & Environment 41: 1251–1262. 10.1111/pce.13133 29314047

