# Supplementary material for: Seagrasses are most vulnerable to marine heatwaves in tropical zones: local‐scale and broad climatic zone variation in thermal tolerances
Source: New Phytol. 2025 Dec 2;249(6):2835–51. doi: 10.1111/nph.70742 (PMC12917458; doi:10.1111/nph.70742)
Supplement: Supplementary file 1 — Fig. S1 Experimental setup for testing seagrass thermal tolerance under light and dark conditions. Fig. S2 Metabolic rate change (net photosynthesis Q10) below optimum temperature across seagrass species and sites. Table S1 Collection location, date, and coordinates for each seagrass species. Table S2 Chamber volume and orientation for experimental conditions. Table S3 Gross photosynthesis and respiration for seagrass species and locations. Table S4 Whole plant metabolism net photosynthesis derived per day for species and locations. Table S5 Differences in thermal optima between daily plant metabolism and hourly net photosynthesis. Please note: Wiley is not responsible for the content or functionality of any Supporting Information supplied by the authors. Any queries (other than missing material) should be directed to the New Phytologist Central Office. [file NPH-249-2835-s001.pdf]

New Phytologist Supporting Information
Article title: Seagrasses are most vulnerable to marine heatwaves in tropical zones: Local-scale and broad climatic zone variation in thermal tolerances
Authors: Nicole Said, Chanelle Webster, Simone Strydom, Natasha Dunham, Matthew P. Adams, and Kathryn McMahon
Article acceptance date: 24 October 2025

Table S1. For each seagrass species, collection location (and site where appropriate), collection month and year, and GPS coordinates of where plants were collected from.

Species	Collection location	Collection Site	Collection month/year	Latitude	Longitude
	Geraldton		Jun 2022	28.748°S	114.610°E
	Jurien Bay		Jun 2022	30.330°S	115.031°E
	Perth	Shoalwater	Jun 2022	32.272°S	115.690°E
P. sinuosa	Perth	Southern Flats	Apr 2024	32.241°S	115.707°E
	Perth	Garden Island	Apr 2024	32.157°S	115.684°E
	Perth	Woodman	Apr 2024	32.129°S	115.738°E
	Geographe		Aug 2022	33.616°S	115.128°E
P. australis	Shark Bay		Aug 2022	25.824°S	113.463°E
	Perth	Woodman	Jul 2022	32.136°S	115.746°E
	Coral Bay		Sep 2023	22.723°S	113.710°E
A. antarctica	Shark Bay		Aug 2022	25.824°S	113.463°E
	Perth	Shoalwater	Jun 2022	32.272°S	115.690°E
A. griffithii	Perth	Shoalwater	Jun 2022	32.272°S	115.690°E
Z. nigricaulis	Perth	Woodman	Jun 2022	32.136°S	115.746°E
	Coral Bay		Sep 2023	23.168°S	113.762°E
H. ovalis	Shark Bay		Mar 2024	26.031°S	113.554°E
	Perth	Shoalwater	Aug 2022	32.272°S	115.690°E

Table S2. Chamber volume and orientation for experimental conditions for each species. Note further 110 ml was added to volume calculation based on water within tubing connecting chambers and pump.

Species	Chamber volume (ml)	Chamber orientation
P. sinuosa	850	Vertical
P. australis	1340	Vertical
A. antarctica	1340	Vertical
A. griffithii	1340	Vertical
Z. nigricaulis	290	Vertical
H. ovalis	320	Horizontal

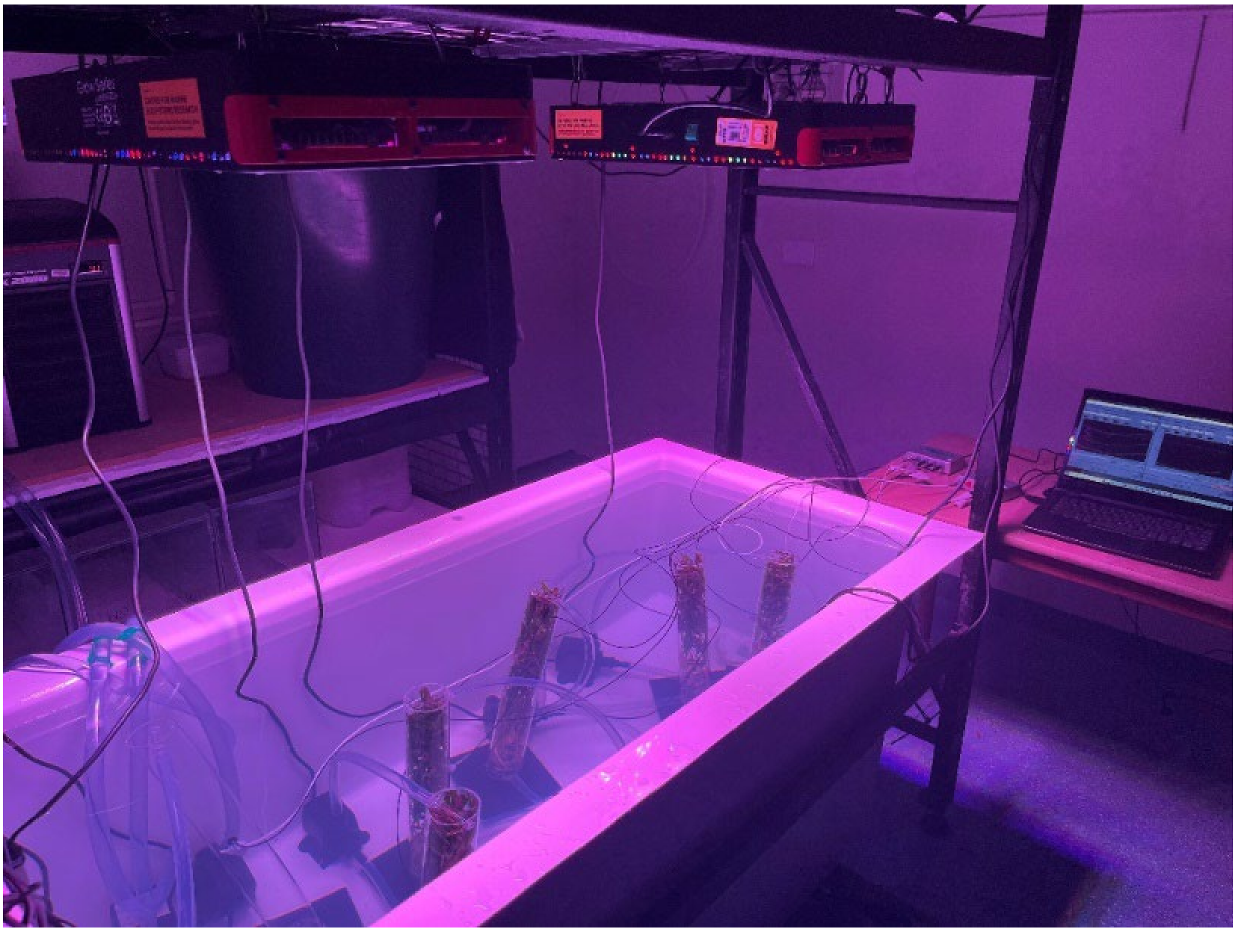

Figure S1. Experimental set up for testing seagrass thermal tolerance under light and dark conditions. The species being assessed in this photo is *Amphibolis antarctica* with 55 cm length cylinders in vertical position in water column. (n=5).

Table S3. Gross photosynthesis: T_{opt}, CT_{max}, Q_{10}, and maximum net productivity (GP_{max}) at T_{opt} from Yan & Hunt model, and for respiration: the maximum respiration rate and at which temperature this rate was observed, and Q_{10} for seagrass species, locations and sites assessed. It is important to note that T_{opt} for gross photosynthesis does not consider the whole plant budget and is therefore not an appropriate indicator in a management context. *Cockburn Sound populations: 1. Southern Flats, 2. Garden Island, 3. Woodman. T_{opt} and CT_{max} are reported to the nearest 0.5 ◦C.

			**Gross Photosynthesis**				**Respiration**			**AG:BG biomass ratio**
**Species**	**Location**	**Site**	T_{opt} (◦C)	CT_{max} (◦C)	Q_{10}	GP_{max}, at T_{opt} (mg O_2 . g^{-1} AG DW . h^{-1})	Temperature at maximum R (°C)*	Q_{10}	Maximum R (mg O_2 . g^{-1} DW . h^{-1})	
P. sinuosa	Geraldton		30.0 ± 0.38	42.0 ± 0.70	1.9 ± 0.17	2.88 ± 0.10	38	1.6 ± 0.1	-0.67 ± 0.05	0.6 ± 0.05
	Jurien Bay		31.5 ± 0.20	41.5 ± 0.39	2.0 ± 0.09	2.94 ± 0.06	38	1.7 ± 0.1	-0.65 ± 0.07	0.6 ± 0.06
	Perth	Shoalwater	28.5 ± 0.23	42.5 ± 0.40	1.7 ± 0.08	3.91 ± 0.06	38	1.9 ± 0.1	-0.68 ± 0.02	0.3 ± 0.03
	Perth	Cockburn Sound 1	31.5 ± 0.19	40.0 ± 0.29	2.3 ± 0.12	2.53 ± 0.06	38	1.6 ± 0.1	-0.38 ± 0.02	0.4 ± 0.05
	Perth	Cockburn Sound 2	32.0 ± 0.38	40.0 ± 0.68	2.4 ± 0.24	3.22 ± 0.16	38	1.7 ± 0.1	-0.46 ± 0.03	0.3 ± 0.02
	Perth	Cockburn Sound 3	31.0 ± 0.32	40.0 ± 0.47	2.0 ± 0.18	2.72 ± 0.10	38	1.7 ± 0.1	-0.39 ± 0.01	0.3 ± 0.03
	Geographe Bay		30.5 ± 0.02	39.5 ± 0.03	2.1 ± 0.10	2.42 ± 0.05	39	1.5 ± 0.1	-0.32 ± 0.02	0.3 ± 0.02
P. australis	Shark Bay		31.5 ± 0.24	43.0 ± 0.48	1.7 ± 0.09	2.72 ± 0.06	39	1.5 ± 0.3	-0.37 ± 0.01	0.3 ± 0.01
	Perth		28.5 ± 0.27	41.0 ± 0.41	1.7 ± 0.09	4.05 ± 0.09	38	1.8 ± 0.1	-0.75 ± 0.01	0.3 ± 0.03
A. antarctica	Coral Bay		25.0 ± 0.95	34.5 ± 0.37	NA	1.39 ± 0.10	39	1.6 ± 0.1	-1.0 ± 0.07	1.6 ± 0.17
	Shark Bay		30.5 ± 0.29	40.5 ± 0.38	1.9 ± 0.07	1.70 ± 0.05	33*	1.5 ± 0.6	-0.53 ± 0.01	2.2 ± 0.08
	Perth		28.5 ± 0.43	44.0 ± 0.80	1.5 ± 0.08	1.31 ± 0.03	39	1.6 ± 0.1	-0.70 ± 0.03	3.9 ± 0.16
A. griffithii	Perth		29.5 ± 0.33	43.0 ± 0.70	1.6 ± 0.10	2.11 ± 0.05	36*	1.7 ± 0.1	-0.69 ± 0.08	2.1 ± 0.14
Z. nigricaulis	Perth		25.0 ± 0.75	42.0 ± 1.40	1.3 ± 0.13	2.86 ± 0.09	36	1.7 ± 0.1	-1.2 ± 0.07	1.5 ± 0.16
H. ovalis	Coral Bay		32.5 ± 0.22	42.5 ± 0.18	2.1 ± 0.18	9.34 ± 0.20	42	1.5 ± 0.1	-1.8 ± 0.02	0.7 ± 0.05
	Shark Bay		35.0 ± 0.18	45.0 ± 0.33	2.1 ± 0.08	2.53 ± 0.05	42	1.6 ± 0.1	-1.0 ± 0.02	1.2 ± 0.14
	Perth		32.5 ± 0.29	44.5 ± 0.28	1.7 ± 0.06	3.82 ± 0.09	43	1.5 ± 0.4	-1.8 ± 0.06	1.3 ± 0.10

All respiration rates increased with temperature and had the highest respiration at the maximum temperature tested, except rows with *.

Table S4. Whole plant metabolism net photosynthesis derived per day (ratio of P:R based on summer and winter daylight hours for each climatic zone; see methods Section 2.5) from Yan & Hunt model, for T_{opt}, CT_{max}, and NP_{max} for both summer and winter. *Cockburn Sound populations: 1. Southern Flats, 2. Garden Island, 3. Woodman. T_{opt} and CT_{max} are reported to the nearest 0.5 ◦C.

Species	Location	Site	T_{opt} (◦C)		CT_{max} (◦C)		NP_{max} (mg O_2 . g^{-1} DW . d^{-1})	
			Summer	Winter	Summer	Winter	Summer	Winter
P. sinuosa	Geraldton		28.5 ± 0.5	29.0 ± 0.6	36.0 ± 0.2	34.0 ± 0.3	7.40 ± 0.43	2.70 ± 0.29
	Jurien Bay		31.0 ± 0.3	NA	36.5 ± 0.1	NA	7.63 ± 0.38	NA
	Perth	Shoalwater	25.5 ± 0.3	25.0 ± 0.4	34.5 ± 0.1	32.0 ± 0.2	7.01 ± 0.19	3.10 ± 0.16
	Perth	Cockburn Sound 1	31.5 ± 0.3	NA	36.0 ± 0.1	NA	3.15 ± 0.19	NA
	Perth	Cockburn Sound 2	31.5 ± 0.5	NA	35.0 ± 0.2	NA	1.72 ± 0.24	NA
	Perth	Cockburn Sound 3	30.0 ± 0.5	NA	35.5 ± 0.2	NA	3.32 ± 0.22	NA
	Geographe Bay		30.0 ± 0.5	NA	35.0 ± 0.3	NA	2.11 ± 0.17	NA
P. australis	Shark Bay		30.5 ± 0.4	NA	37.0 ± 0.2	NA	3.22 ± 0.18	NA
	Perth		27.0 ± 0.5	27.0 ± 0.5	34.5 ± 0.2	32.5 ± 0.3	7.05 ± 0.28	2.67 ± 0.25
A. antarctica	Coral Bay		25.5 ± 0.9	24.5 ± 1.1	35.5 ± 0.3	33.5 ± 0.4	14.19 ± 0.90	8.21 ± 0.77
	Shark Bay		29.0 ± 0.5	28.5 ± 0.9	36.0 ± 0.2	34.0 ± 0.5	5.48 ± 0.29	2.05 ± 0.26
	Perth		25.0 ± 0.5	24.0 ± 0.8	34.5 ± 0.2	31.0 ± 0.5	5.60 ± 0.24	1.49 ± 0.24
A. griffithii	Perth		25.0 ± 0.5	22.5 ± 0.8	35.5 ± 0.2	31.5 ± 0.4	8.71 ± 0.30	3.53 ± 0.27
Z. nigricaulis	Perth		21.0 ± 0.9	19.0 ± 1.0	31.0 ± 0.4	26.5 ± 0.6	9.72 ± 0.65	3.20 ± 0.59
H. ovalis	Coral Bay		30.5 ± 0.4	29.5 ± 0.5	38.0 ± 0.2	36.0 ± 0.3	19.54 ± 0.83	8.79 ± 0.69
	Shark Bay		NA	NA	NA	NA		NA
	Perth		32.5 ± 0.4	NA	38.0 ± 0.2	NA	7.67 ± 0.58	NA

Scaling up physiological thermal estimates using daily plant metabolism

When scaling up hourly rates of net photosynthesis (NP; mg O_2. g DW^{-1}. hr^{-1}; Table 2) into daily rates of plant metabolism across an entire 24 hour period (day and night), in a standard summer day and winter day, the T_{opt} for daily plant metabolism was either equal to or lower than the T_{opt} based on NP hourly rates across both summer and winter. Where T_{opt} for daily plant metabolism values were lower in summer the difference in T_{opt} (from hourly NP) ranged from 0 °C to 2 °C, and in winter 0 °C to 4.5 °C. There was one exception where the T_{opt} values for *Halophila ovalis* in the Perth location were higher for daily plant metabolism by 0.5 °C.

Table S5. Difference in thermal optima (T_{opt}) for daily plant metabolism (mg O_2. g DW^{-1}. d^{-1}) for both summer and winter, in comparison to hourly net photosynthesis rates (mg O_2. g DW^{-1}. hr^{-1}). Daily plant metabolism (24 hours) for summer (S) and winter (W) are defined by daylight hours with the ratio of NP:R changing across seasons and location as per Section 2.5. Coloured cells represent the T_{opt} of daily plant metabolism being higher, equal, or lower than the T_{opt} of hourly net photosynthesis rates. Where T_{opt} was either higher or lower the difference in T_{opt} has is written in the cell. Blank cells are where species were not collected at a location. NA = model could not be fitted. All data for daily plant metabolism (including T_{opt}, CT_{max} and NP_{max} at T_{opt}) can be found in Table S4. *Cockburn Sound populations: 1. Southern Flats, 2. Garden Island, 3. Woodman.

							**Higher**		**Equal**		**Lower**	
**Location**	**Seagrass species**											
	Posidonia sinuosa		*Posidonia australis*		*Amphibolis antarctica*		*Amphibolis griffithii*		*Zostera nigricaulis*		*Halophila ovalis*	
	S	W	S	W	S	W	S	W	S	W	S	W
Coral Bay						2.5 °C					1 °C	2 °C
Shark Bay				NA	1 °C	1.5 °C					NA	NA
Geraldton												
Jurien Bay		NA										
Perth Shoalwater	1 °C	1.5 °C			1 °C	2 °C	2 °C	4.5 °C			0.5 °C	NA
Perth Cockburn Sound*		NA								3.5 °C		
Geographe Bay		NA										

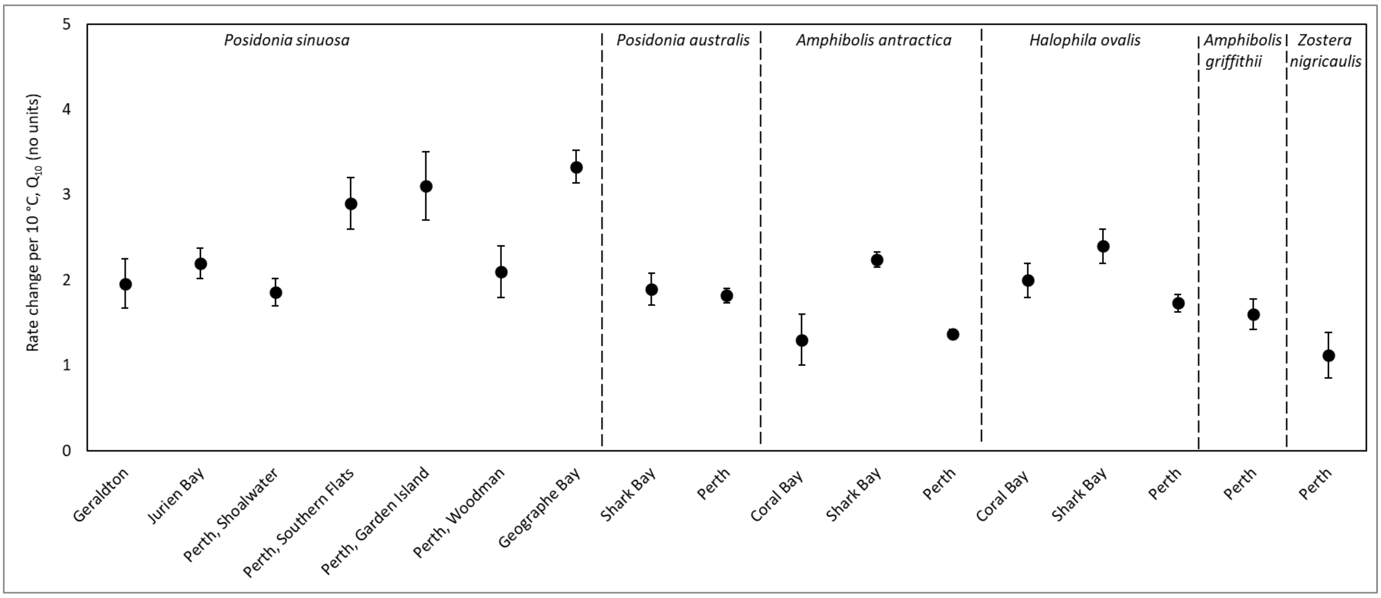

Figure S2. The rate of metabolic change (net photosynthesis Q_{10}) below the optimum temperature for seagrass species across and within location and sites where appropriate.
